# Autophagy repression by antigen and cytokines shapes mitochondrial, migration and effector machinery in CD8 T cells

**DOI:** 10.1038/s41590-025-02090-1

**Published:** 2025-02-27

**Authors:** Linda V. Sinclair, Tom Youdale, Laura Spinelli, Milica Gakovic, Alistair J. Langlands, Shalini Pathak, Andrew J. M. Howden, Ian G. Ganley, Doreen A. Cantrell

**Affiliations:** 1 Division of Cell Signalling and Immunology, School of Life Sciences, University of Dundee, Dundee, UK; 2 National Phenotypic Screening Centre, School of Life Sciences, University of Dundee, Dundee, UK; 3 MRC PPU, School of Life Sciences, University of Dundee, Dundee, UK

**Keywords:** Cytotoxic T cells, Autophagy

## Abstract

Autophagy shapes CD8 T cell fate; yet the timing, triggers and targets of this process are poorly defined. Herein, we show that naive CD8 T cells have high autophagic flux, and we identify an autophagy checkpoint whereby antigen receptor engagement and inflammatory cytokines acutely repress autophagy by regulating amino acid transporter expression and intracellular amino acid delivery. Activated T cells with high levels of amino acid transporters have low autophagic flux in amino-acid-replete conditions but rapidly reinduce autophagy when amino acids are restricted. A census of proteins degraded and fueled by autophagy shows how autophagy shapes CD8 T cell proteomes. In cytotoxic T cells, dominant autophagy substrates include cytolytic effector molecules, and amino acid and glucose transporters. In naive T cells, mitophagy dominates and selective mitochondrial pruning supports the expression of molecules that coordinate T cell migration and survival. Autophagy thus differentially prunes naive and effector T cell proteomes and is dynamically repressed by antigen receptors and inflammatory cytokines to shape T cell differentiation.

## Abstract (web summary)

Sinclair et al. combine quantitative proteomics and an autophagy flux reporter to map autophagy substrates and triggers during CD8 T cell differentiation. Proteins degraded and fueled by autophagy in naïve and effector T cells are identified and it is revealed that the regulation of amino acid transport allows antigen receptors and inflammatory cytokines to repress autophagy flux to shape T cell differentiation.

## Main

CD8 T lymphocytes shape transcriptional outputs by regulating protein synthesis and degradation. Naive and memory T cells have low levels of protein synthesis and T cell clonal expansion, and cytotoxic T cell (CTL) differentiation is driven by increases in protein synthesis that implement T cell transcriptional programs. Protein synthesis requires amino acids that are obtained from the environment by membrane amino acid transporters or supplied by autophagy, which degrades intracellular proteins to recycle amino acids. Autophagy is critical for naive CD8 T cell survival^1–7^ and memory cell formation^7–10^. Autophagy also restrains CTL function, and its loss promotes CD8 T cell anti-tumor immunity^11,12^. Given the importance of autophagy for CD8 T cells, it is essential to understand how this process is controlled and what proteins are degraded and fueled by autophagy in different CD8 T cell populations. Initial studies proposed that immune activation switches on autophagy^1,2,13–15^. These conclusions were based on measuring autophagosome levels or quantitating expression of autophagy markers such as microtubule-associated protein 1 light chain 3 beta (MAP1LC3b)^16–20^. By contrast, experiments with dynamic autophagy flux reporters observed low autophagy in proliferating T cells and high autophagy in memory T cells^7^. To resolve these discrepancies, the present study uses mass spectrometry and a dynamic autophagy reporter in tandem to comprehensively quantify autophagy machinery and autophagy flux as T cells differentiate. Our findings reveal that autophagy flux is high in naive T cells but rapidly repressed by antigen receptor engagement. Autophagy machinery expression is high in effector cells but autophagy flux is dynamic and repressed by pro-inflammatory cytokines but not by cytokines that promote the formation of memory T cells. The control of amino acid transporter expression and intracellular amino acid delivery by antigen receptors and cytokines is identified as a critical autophagy checkpoint for T cells. A proteomic census also maps autophagy substrates and proteins fueled by autophagy in naive and effector CD8 T cells, which explains why precise regulation of autophagy allows CD8 T cells to initiate and curtail effector function.

## Results

### Immune-activated T cells accumulate autophagy machinery but repress autophagy flux

**Fig. 1:**
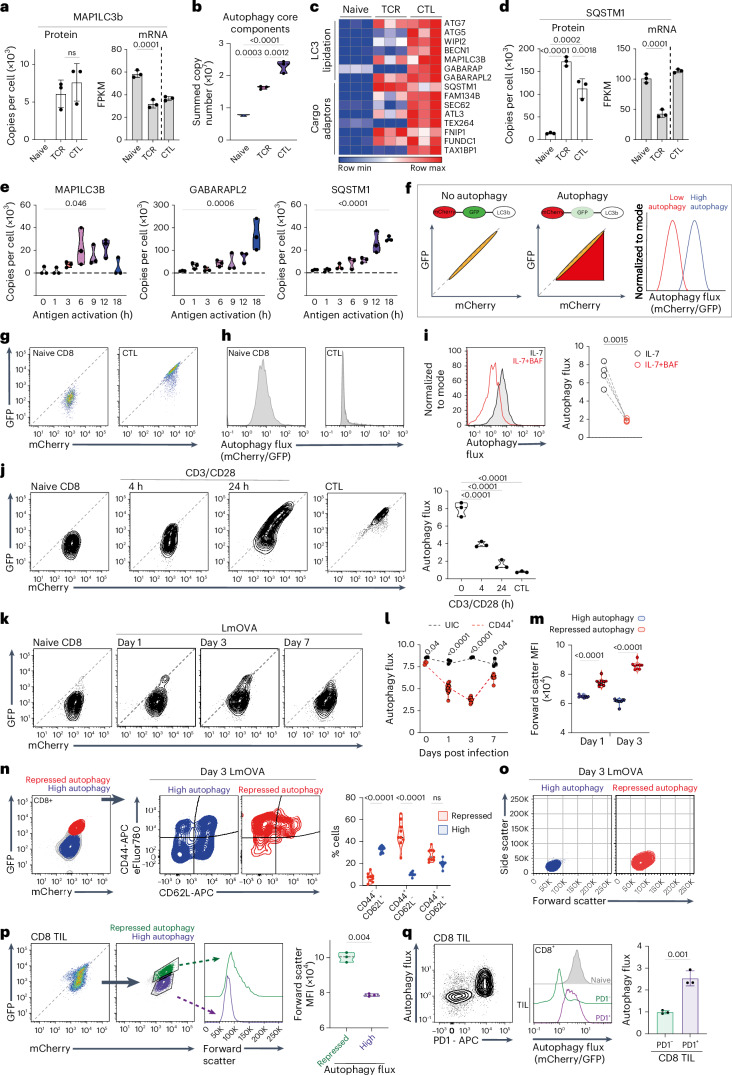
Immune-activated T cells repress autophagy flux. **a**–**d**, Data are from naive, 24 h antigen-activated P14-CD8 T cells (TCR) and IL-2-maintained CTLs. **a**, MAP1LC3B protein copies per cell and mRNA (fragments per kilobase million (FPKM)). **b**, Summed copies of core autophagy proteins^72^ (Source Data Fig. 1). **c**, Heatmap of autophagy machinery. **d**, SQSTM1 protein copies and mRNA. **e**, MAP1LC3B, GABARAPL2 and SQSTM1 protein copies from naive and antigen-activated OT-1 CD8 T cells. **f**, Schematic flow cytometry data of cells expressing mCherry–GFP–LC3b autophagy reporters. Equivalent GFP and mCherry fluorescence indicates no autophagy; GFP fluorescence quenching indicates high autophagy. mCherry/GFP fluorescence-derived parameters plotted as histograms. **g**, GFP/mCherry fluorescence profiles of mCherry–GFP–LC3b CD8 T cells: naive (left) and CTL (right). **h**, Autophagy flux in mCherry–GFP–LC3b CD8 cells: naive (left) and CTL (right). **i**, Autophagy flux in mCherry–GFP–LC3b naive CD8 T cells maintained in IL-7 ± bafilomycin A (BAF, 200 nM) for 5 h. **j**, GFP/mCherry fluorescence and autophagy flux in naive, 4 h or 24 h, CD3/CD28-activated mCherry–GFP–LC3b CD8 T cells and CTLs. **k**–**o**, Autophagy reporter mice were infected with OVA expressing *Listeria monocytogenes* (LmOVA). **k**, GFP/mCherry fluorescence of splenic CD8 cells from uninfected (naive) and day 1, 3 and 7 LmOVA-infected mCherry–GFP–LC3b mice. **l**, Autophagy flux of CD44^+^ CD8 T cells from day 1, 3 and 7 LmOVA-infected or uninfected mCherry–GFP–LC3b mice. **m**, Forward-scatter mean fluorescence intensity (MFI) of mCherry–GFP–LC3b CD8 T cells with high or repressed autophagy on day 1 and day 3 post LmOVA infection. **n**, CD62L/CD44 levels on mCherry–GFP–LC3b CD8 T cells on day 3 post LmOVA infection. **o**, Forward-scatter vs side-scatter profiles of GFP–mCherry–LC3b CD8 T cells with high or repressed autophagy on day 3 post LmOVA infection. **p**,**q** Flow cytometry analysis of tumor-infiltrating CD8 T cells (CD8-TILs) from MC38 tumors engrafted in GFP–mCherry–LC3b mice. **p**, GFP/mCherry fluorescence of mCherry–GFP–LC3b CD8-TILs (left) and forward-scatter analysis comparing high and repressed autophagy CD8-TILs (right). **q**, CD8-TIL autophagy flux and PD1 expression (left); autophagy flux of PD1^−^ and PD1^+^ CD8-TIL vs naive CD8 T cells (right). Proteomic data in **a**–**d** are from ref. ^21^ or ref. ^22^; mRNA data are from ref. ^73^. Data are from *n* = 3 (**a**–**e**,**j**), *n* = 6 (**g**,**h**), *n* = 4 (**i**), *n* = 9 (**k**–**o**) and *n* = 3 (**p**,**q**) mice or biological replicates per condition. Flow plots show representative data. Bar charts and violin plot data indicate biological replicates. Error bars, s.d. Source data

**Extended Data Fig. 1:**
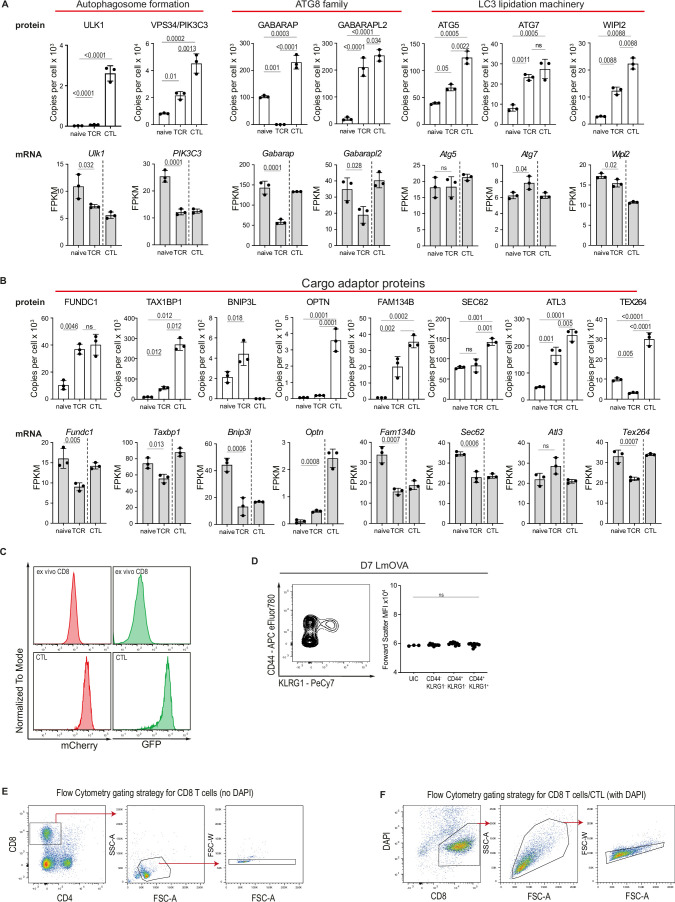
Expression of autophagy related proteins during immune activation. Related to Fig. 1. **A**, **B** Quantitative proteomics data showing mean protein copy number per cell and RNASeq data shown as FPKM from naïve, 24 h TCR activated P14 CD8 T cells and IL-2 maintained effector CD8 T cells (CTL) for **A)** ULK1, VPS34(PIK3C3), GABARAP, GABARAPL2, ATG5, ATG7 and WIPI2 and **B)** FUNDC1, TAX1BP1, BNIP3L, OPTN, FAM134B, SEC63, ATL3 and TEX264. **C** Representative flow cytometry data showing mCherry (left) and GFP (right) fluorescence profiles of ex vivo, naïve CD8 cells and IL-2 maintained effector CD8 T cells (CTL). **D** GFP:mCherry:LC3b autophagy reporter mice were infected with attenuated OVA expressing *Listeria monocytogenes* (LmOVA). Representative flow cytometry of CD44 and KLRG1 expression (left) and matched FSc MFI (right) from CD8 T cells on D7 post LmOVA infection. **E, F** Representative gating strategies for identification of single CD8 T cells with or without DAPI staining for viability. Proteomic data are from Howden et al.^21^ or Immpres.co.uk^22^. mRNA data are from Spinelli et al.^73^. Data are from a minimum of 3 experimental repeats. Bar charts data indicate biological replicates. Error bars represent means +/-SD.

To assess whether immune activation changes the abundance of autophagy machinery, we examined mass spectrometry data that quantitatively analyzed T cell proteomes^21,22^. These data show that the ATG8-family protein MAP1LC3b is not detected in naive T cells but is abundant in antigen-activated T cells and CTLs (Fig. 1a). CD8 T cells also increase the abundance of ATG8 proteins GABARAP and GABARAPL2 as they differentiate into CTLs (Extended Data Fig. 1a). The abundance of other core autophagy components also increases as CD8 T cells differentiate (Fig. 1b,c, Extended Data Fig. 1a and Source Data Fig. 1). Sequestosome-1 (p62/SQSTM1), an autophagy adaptor that targets proteins for degradation, accumulates in immune-activated cells (Fig. 1d), as do cargo adaptors for mitochondrial autophagy (mitophagy) and endoplasmic reticulum autophagy (ER-phagy) (Fig. 1c and Extended Data Fig. 1b). Autophagy protein accumulation occurs rapidly, within 3–6 h of antigen exposure (Fig. 1e), and does not correlate with an increased abundance of corresponding mRNA (Fig. 1a,d and Extended Data Fig. 1a,b).

Cells with high autophagic flux constantly degrade autophagy machinery; autophagy proteins would be in low abundance when autophagy rates are high but would accumulate when autophagy is repressed. A low abundance of MAP1LC3b and GABARAPs in naive T cells and their accumulation in activated CD8 T cells could thus reflect that naive T cells exhibit high autophagy but repress autophagy as they respond to immune activation. To explore this hypothesis, we used an autophagy flux reporter that incorporates the normalization of autolysosome levels to accurately measure autophagy flux in primary tissues^23,24^. In this model, an mCherry–GFP–Map1lc3b (mCherry–GFP–LC3b) fusion protein is expressed ubiquitously from the *ROSA26* locus. When autophagy initiates, tandem-tagged LC3b is recruited into autolysosomes, where low luminal pH quenches GFP fluorescence while mCherry fluorescence remains stable. Increased autophagic flux is visualized and quantified by measuring GFP fluorescence quenching normalized to mCherry fluorescence. Flow cytometry shows that cells not undergoing autophagy have linear GFP vs mCherry fluorescence profiles whereas cells undergoing autophagy have quenched (lower) GFP vs mCherry signals (Fig. 1f).

Figure 1g and Extended Data Fig. 1c show that naive CD8 T cells and in-vitro-generated CTLs expressing the LC3b reporter have a strong mCherry signal. However, there is a quenching of GFP fluorescence in naive CD8 T cells compared with a high linear correlation of GFP/mCherry signals in CTLs (Fig. 1g,h). This GFP quenching pattern indicates that the autophagy reporter is in acidic compartments in naive T cells but not in CTLs. Bafilomycin A1, which inhibits lysosomal V-ATPases, raises lysosomal pH and prevents lysosome–autophagosome fusion^25,26^. High levels of autophagy flux in naive T cells are reduced in bafilomycin-treated cells (Fig. 1i). In further experiments, naive CD8 T cells expressing the autophagy reporter were activated in vitro with CD3/CD28 antibodies and cellular GFP/mCherry fluorescence profiles analyzed over time. Figure 1j shows that a population of cells with no GFP quenching emerge within a few hours of CD3/CD28 activation. After 24 h activation, the majority of cells had linear GFP/mCherry fluorescence profiles (Fig. 1j). Hence, high autophagy flux in naive T cells is rapidly repressed by immune activation. The autophagic flux reductions detected within a few hours of immune activation coincide with the timing of accumulation of ATG8 proteins and cargo adaptors in antigen-activated CD8 T cells (compare Fig. 1j vs 1e).

We also examined autophagy in CD8 T cells activated in vivo when mCherry–GFP–LC3b reporter mice were immunized with *Listeria monocytogenes*. The data identify a population of CD8 T cells with reduced autophagy flux in day 1 and day 3 *Listeria-*infected mice and show that autophagy flux in CD8 T cells returns to high levels similar to naive T cells by day 7 (Fig. 1k,l). CD8 T cells with repressed autophagy have increased cell size (Fig. 1m); the majority are CD44^+^CD62L^−^, corresponding to effector cells, but include some CD44^+^CD62L^+^ cells, corresponding to memory precursors (Fig. 1n)^27^. In day 3 *Listeria*-infected mice, most effector-phenotype cells had repressed autophagy but a small population of non-blasted, CD44^+^CD62L^−^ cells had high autophagy (Fig. 1n,o). By day 7 of *Listeria* infection, all CD8 T cells were small, non-blasted cells with high autophagy flux (Fig. 1k,l and Extended Data Fig. 1d). We also examined autophagy in tumor-infiltrating CD8 T cells. mCherry–GFP–LC3b reporter mice were engrafted with MC38 colon-adenocarcinoma cells, and autophagy was examined in tumor-infiltrating CD8 T cells. Fig. 1p shows that CD8 T cells infiltrating tumors are heterogeneous for autophagy flux; notably, a population of PD1^+^ CD8 T cells shows high autophagy flux and a population of PD1^−^ CD8 T cells exhibits repressed autophagy (Fig. 1q). Cells with high autophagy were small whereas repressed autophagy cells were large (Fig. 1p). Collectively, these analyses of quiescent and immune-activated T cells in vitro and in vivo demonstrate high levels of autophagic flux in naive T cells, which is acutely and transiently repressed by immune activation.

### Pro-inflammatory cytokines repress autophagy flux

**Fig. 2:**
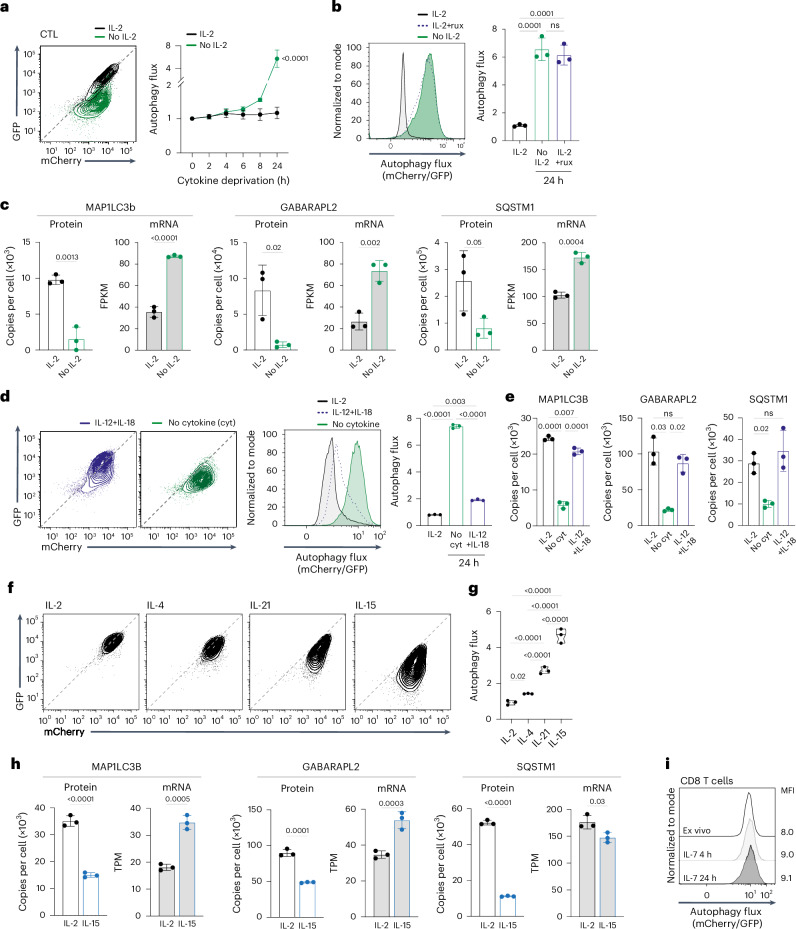
Pro-inflammatory but not memory cytokines repress autophagy flux. **a**, Representative GFP/mCherry fluorescence profiles from mCherry–GFP–LC3b autophagy reporter CTLs maintained with and without IL-2 for 24 h (left) and autophagy flux over the expanded time course (right). **b**, Autophagy flux in mCherry–GFP–LC3b CTLs maintained with and without IL-2 or the JAK inhibitor ruxolitinib (rux, 1 µM) for 24 h (left) and mean autophagy flux (right). **c**, MAP1LC3B, GABARAP2L and SQSTM1 mean protein copies per cell and mRNA (shown as FPKM) from CTLs maintained with and without IL-2 over 24 h. **d**, GFP/mCherry fluorescence profiles (left), autophagy flux histogram (center) the mean autophagy flux (mCherry/GFP) values (right) from mCherry–GFP–LC3b CTLs maintained with IL-2, IL-12 + IL-18 or no cytokine for 24 h. **e**, MAP1LC3B, GABARAP2L and SQSTM1 protein copies per cell from CTLs maintained with IL-2, IL-12 + IL-18 or no cytokine for 24 h. **f**, GFP/mCherry fluorescence profiles of antigen-activated mCherry–GFP–LC3b CD8 T cells expanded with IL-2 (CTL) or switched into IL-4 or IL-21 for 24 h, or expanded with IL-15 (memory-like). **g**, Mean autophagy flux from mCherry–GFP–LC3b CTLs expanded with IL-2 or switched into IL-4 or IL-21 for 24 h, or antigen-activated CD8 T cells expanded with IL-15 (memory-like). **h,** MAP1LC3B, GABARAP2L and SQSTM1 protein copies per cell and mRNA (shown as transcripts per million (TPM)) from antigen-activated CD8 T cells expanded with IL-2 or IL-15. **i,** Autophagy flux in mCherry–GFP–LC3b CD8 T cells, either ex vivo or maintained in IL-7 for 4 h or 24 h; corresponding MFI is indicated. Data in **c**, **e**, and **h** are derived from refs. ^22,73,74^, respectively. Data are from *n* = 3 mice or biological replicates per condition. Flow plots show representative data. Bar charts and violin plot data indicate biological replicates. Error bars, s.d.

**Extended Data Fig. 2:**
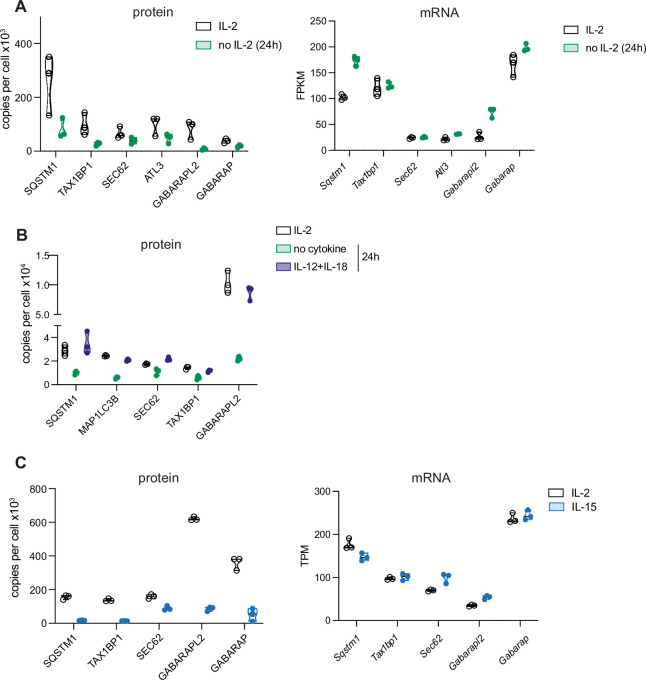
Expression of autophagy related proteins in response to pro-inflammatory or memory cytokines. Related to Fig. 2. **A** Quantitative proteomics data of the indicated proteins showing mean protein copy number per cell (left) and RNASeq data shown as fragments per kilobase of transcript per million mapped reads (FPKM; right) from CTL maintained with or without IL-2 over 24 h. Data are from Spinelli et al.^73^. **B** Quantitative proteomics data of the indicated proteins showing mean protein copy number per cell from CTL maintained with IL-2, with IL-12+IL-18, or no cytokine for 24 h. Data are available from Immpres.co.uk^22^. **C** Quantitative proteomics data of the indicated proteins showing mean protein copy number per cell (left) and RNASeq data shown as transcripts per million (TPM; right) from CD8 T cells expanded with IL-2 (CTL) or expanded with IL-15 (memory-like). Data are from Marchingo et al.^74^. Data points in bar charts are indicative of biological replicates. Error bars represent the mean +/- SD.

In-vitro-differentiated CTLs have low autophagy flux (Fig. 1g,j), and an important question is whether this is an intrinsic state or directed by the cytokines used to drive CTL differentiation. To explore this question, we examined how interleukin-2 (IL-2), used to clonally expand and differentiate CTLs, impacted autophagy. Fig. 2a,b shows that CTLs cultured in IL-2 have linear GFP/mCherry autophagy reporter fluorescence consistent with low autophagy flux. However, IL-2-deprived CTLs, or IL-2-maintained CTLs treated with kinase inhibitors to block IL-2 signaling, revert to a profile of quenched GFP/mCherry fluorescence; that is, high autophagy flux. Consistent with increased autophagy, there was decreased expression at the protein but not mRNA level of ATG8 proteins and autophagy adaptors in IL-2-deprived CTLs (Fig. 2c and Extended Data Fig. 2a). Thus, autophagy repression in CTLs is dependent on sustained IL-2 signaling. Further experiments revealed that IL-12 and IL-18, which drive terminal effector CD8 T cell differentiation, also repress autophagy in CTLs, allowing ATG8 proteins and autophagy cargo adaptors to accumulate (Fig. 2d,e and Extended Data Fig. 2b). CTLs co-express IL-2, IL-4 and IL-21 receptors. Fig. 2f,g shows that IL-2 is the most potent autophagy repressor, followed by IL-4 and IL-21 (Fig. 2f,g). By contrast, antigen-activated CD8 T cells that are expanded with IL-15 (which promotes memory T cell differentiation) do not suppress autophagy (Fig. 2f,g). There is also decreased expression of ATG8 proteins and cargo adaptors at the protein but not the mRNA level in IL-15-maintained CD8 T cells, consistent with high autophagy (Fig. 2h and Extended Data Fig. 2c). CD8 T cells maintained in IL-7 also do not repress autophagy (Fig. 2i). Collectively, these data reveal high autophagy flux in naive and memory CD8 T cells and autophagy repression when naive cells respond to antigen. Pro-inflammatory cytokines that drive terminal effector T cell differentiation repress autophagy in antigen-primed CD8 T cells, whereas cytokines that support memory T cells do not.

### Amino acid supply controls autophagy in CD8 T cells

**Fig. 3:**
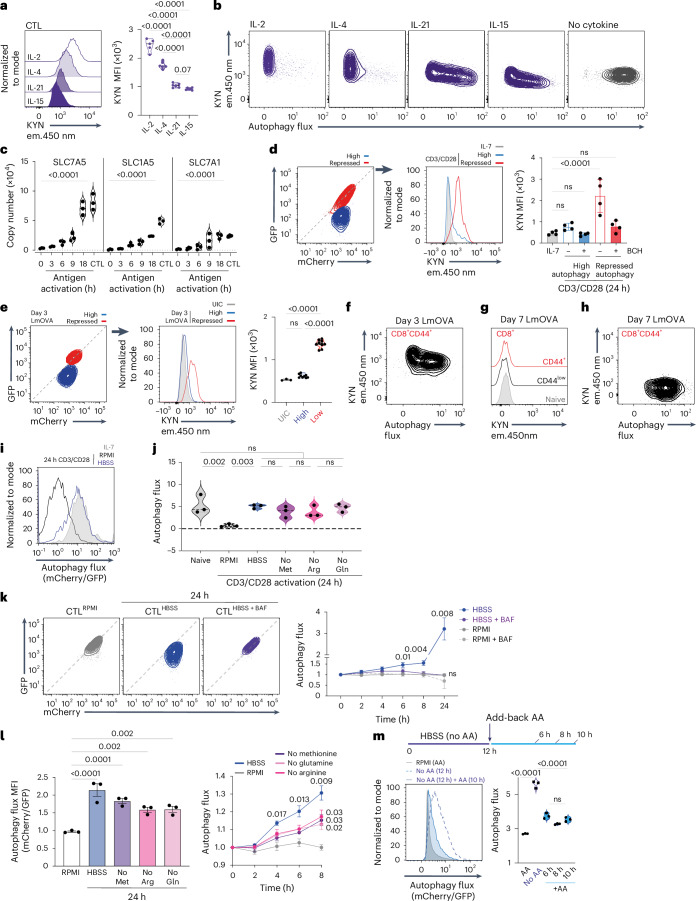
Amino acid supply controls autophagy in CD8 T cells. **a**, Kynurenine (KYN; System L substrate) uptake of antigen-activated CD8 T cells maintained in IL-2, IL-4, IL-21 and IL-15. **b**, Flow cytometry profiles of autophagy flux and kynurenine uptake of antigen-activated CD8 T cells maintained in indicated cytokines for 24 h. **c**, Protein copies per cell of SLC7A5, SLC1A5 and SLC7A1 in CTLs and antigen-activated OT-1 CD8 T cells. **d**, GFP/mCherry fluorescence of 24 h CD3/CD28-activated mCherry–GFP–LC3b CD8 T cells. Left panel, high and repressed autophagy flux is indicated; center panel, kynurenine uptake in IL-7 maintained, or high vs repressed autophagy of CD3/CD28-activated CD8 cells; right panel, kynurenine uptake in presence or absence of BCH (System L competitive substrate). **e**, Left panel, GFP/mCherry fluorescence of mCherry–GFP–LC3b CD8 T cells from day 3 LmOVA-infected mice; high and repressed autophagy populations indicated. Center and right panels, kynurenine uptake of high and repressed autophagy populations. UIC, uninfected control. **f,** Autophagy flux and kynurenine uptake of CD44^+^ mCherry–GFP–LC3b CD8 T cells from day 3 LmOVA-infected mice. **g**, Kynurenine uptake of CD44^+^ and CD44^−^ mCherry–GFP–LC3b CD8 T cells from day 7 LmOVA-infected or control mice. **h**, Autophagy flux and KYN uptake of CD44^+^ mCherry–GFP–LC3b CD8 T cells from day 7 LmOVA-infected mice. **i**, Autophagy flux of IL-7 maintained or 24 h CD3/CD28-activated mCherry–GFP–LC3b CD8 T cells cultured in RPMI or HBSS. **j**, Autophagy flux of mCherry–GFP–LC3b CD8 T cells ex vivo or 24 h CD3/CD28-activated in RPMI/HBSS/RPMI lacking methionine (no Met), arginine (no Arg) or glutamine (no Gln). **k**, Left panel, GFP/mCherry fluorescence of mCherry–GFP–LC3b CTLs maintained in RPMI, or switched into HBSS ± 200 nM BAF for 24 h. Right panel, autophagy flux over experimental time course. **l**, Left panel, autophagy flux in mCherry–GFP–LC3b CTLs maintained in RPMI, switched into HBSS or RPMI lacking methionine, arginine or glutamine for 24 h. Right panel, autophagy flux time course for these conditions. **m**, Left panel, autophagy flux of mCherry–GFP–LC3b CTLs maintained in RPMI or HBSS (no amino acids (no AA)) for 12 h, and HBSS for 12 h followed by RPMI for 10 h. Right panel, autophagy flux in CTLs maintained in RPMI or HBSS for 12 h, followed by RPMI for 6 h, 8 h and 10 h. Proteomic data in **c** are from ref. ^22^. Data are from *n* = 5–6 (**a**,**b**), *n* = 3 (**c**,**i**–**m**), *n* = 4 (**d**), *n* = 3–9 (**e**–**h**) and *n* = 5–6 (**a**,**b**) mice or biological replicates per condition. Flow plots show representative data. Bar charts and violin plot data indicate biological replicates. Error bars, s.d. Data in **k** and **l** were analyzed using two-way ANOVA with Fisher’s least significant difference test.

**Extended Data Fig. 3:**
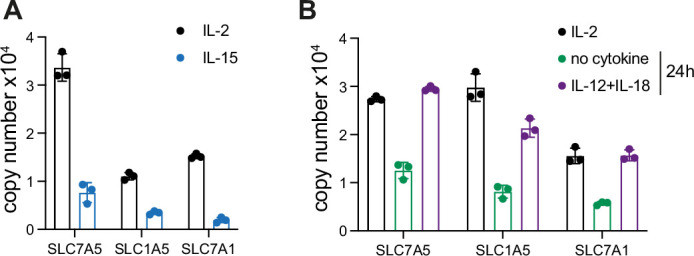
Expression of amino acid transporters in response to pro-inflammatory or memory cytokines. Related to Fig. 3. **A** Quantitative proteomics data showing mean protein copy number per cell of the indicated proteins from CD8 T cells expanded with IL-2 (CTL) or expanded with IL-15 (memory-like). Data are from Marchingo et al.^74^. **B** Quantitative proteomics data showing mean protein copy number per cell of the indicated proteins from CTL maintained with IL-2, with IL-12+IL-18, or no cytokine for 24 h. Data are available from Immpres.co.uk^22^. Data points in bar charts are indicative of biological replicates. Error bars represent the mean +/- SD.

One evolutionarily conserved autophagy stimulus is amino acid starvation^28^. In this context, naive T cells have high autophagy flux and low amino acid transporter levels, whereas antigen-activated T cells with low autophagy flux have high amino acid transporter levels^21,29–32^. Furthermore, IL-15-maintained CD8 T cells with high autophagy flux have low amino acid transport compared to high levels of amino acid transport in IL-2-maintained CTLs^32,33^ (Fig. 3a and Extended Data Fig. 3a,b). The different potency of IL-2, IL-4, IL-21 and IL-15 in the context of autophagy repression also correlates with their ability to induce amino acid transport: IL-2 is most potent, followed by IL-4, IL-21 and IL-15 (Figs. 3a,b and 2f,g). Hence, stimuli that drive high amino acid transport capacity repress autophagy.

Importantly, the timing of autophagy flux repression coincides with the upregulation of amino acid transporters in antigen-activated CD8 T cells (Figs. 1j and 3c). Moreover, differences in amino acid transport underpin autophagic flux heterogeneity in immune-activated CD8 T cells. At 24 h, the majority of activated cells showed linear GFP–mCherry fluorescence, indicating repressed autophagy, but some cells retained quenched GFP fluorescence (Figs. 1j and 3d). Cells with high levels of autophagic flux have low amino acid transport, whereas cells with repressed autophagy have high amino acid transport (Fig. 3d). Importantly, autophagy repression in CD8 T cells activated in vivo also correlates with their amino acid transport capacity. Fig. 3e shows amino acid transport and autophagy flux of CD8 T cells from day 3 *Listeria*-infected mice. CD8 T cells with repressed autophagy have high amino acid transport, whereas antigen-experienced CD8 T cells with high autophagy flux show low amino acid transport (Fig. 3e,f). All antigen-experienced CD8 T cells isolated from day 7 *Listeria*-infected mice have low amino acid transport and high autophagy flux, similar to naive T cells (Fig. 3g,h). Hence, the mechanism that underpins the ability of immune-activated T cells to repress autophagy is linked with the upregulation of amino acid transport to allow intracellular sensing of imported amino acids.

To test a causal link between amino acid supply and autophagy, we examined whether amino-acid-deprived, immune-activated CD8 T cells could repress autophagy. Fig. 3i,j shows that immune-activated T cells cannot repress autophagy unless they have an extracellular amino acid supply; moreover, deprivation of a single amino acid also prevents autophagy repression in response to immune activation. Furthermore, IL-2-maintained CTLs that are switched from amino-acid-replete to amino-acid-free media or media depleted of a single amino acid rapidly increase autophagy (Fig. 3k,l). When amino-acid-deprived CTLs are treated with bafilomycin, autophagy is repressed (Fig. 3k). Re-addition of amino acids to amino-acid-deprived CTLs also re-establishes autophagy repression (Fig. 3m). Autophagy repression in CTLs is thus dynamic and dependent on sustained amino acid supply.

### CTLs control autophagy by VPS34-dependent and AMPK/mTORC1-independent pathways

**Fig. 4:**
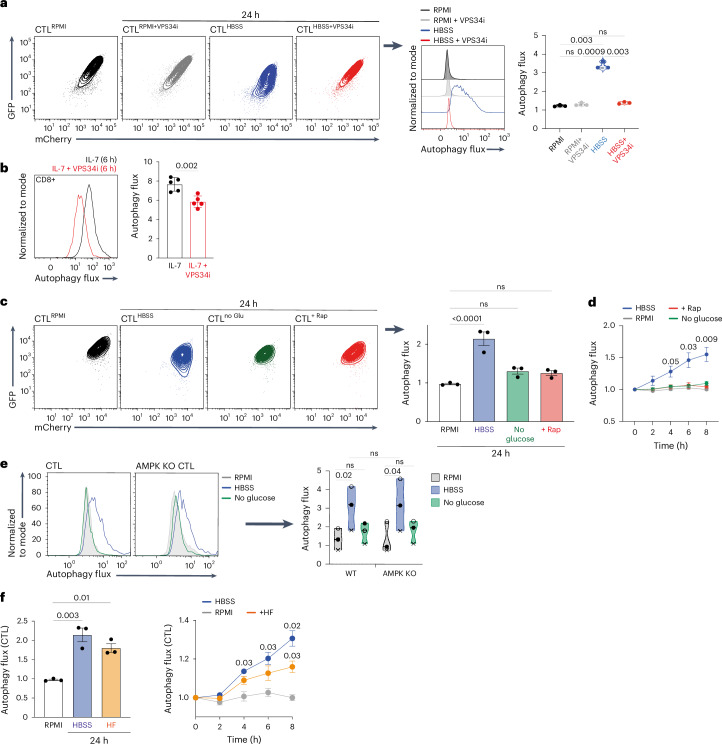
VPS34-dependent and AMPK and mTORC1-independent autophagy in CD8 T cells. **a**, mCherry/GFP flow cytometry plots of mCherry–GFP–LC3b CTLs maintained in the presence of full amino acids (RPMI) ± VPS34 inhibitor (VPS34i) or depleted of amino acids (in HBSS) ± VPS34i for 24 h (left panels). Right panels show the corresponding autophagy flux histogram and means. **b**, Autophagy flux histogram (left) and the mean autophagy flux (right) of naive mCherry–GFP–LC3b CD8 T cells maintained in IL-7 ± VPS34i for 6 h. **c**, Representative mCherry/GFP plots (left) and corresponding mean autophagy flux (right) of mCherry–GFP–LC3b CTLs maintained in the presence of full amino acids (RPMI), depleted of amino acids (HBSS), switched into glucose-free RPMI (no Glu) or treated with rapamycin (Rap, 20 nM) for 24 h. **d**, Autophagy flux values over the expanded time course of mCherry–GFP–LC3b CTLs treated as in **c**. **e**, Autophagy flux histograms (left) and mean autophagy flux values (right) of CTLs from control (WT) mCherry–GFP–LC3b mice or CD4CreAMPK^fl/fl^ (AMPK KO) mCherry–GFP–LC3b autophagy reporter mice maintained in the presence of full amino acids (RPMI), depleted of amino acids (HBSS) or switched into glucose-free RPMI (no glucose) for 24 h. **f**, Mean autophagy flux values of mCherry–GFP–LC3b CTLs maintained in the presence of full amino acids (RPMI), depleted of amino acids (HBSS) or treated with halofuginone (HF; 100 nM) for 24 h (left panel) and over the expanded time course (right panel). Data are from *n* = 3 mice or experimental replicates per condition. Bar chart and violin plots indicate biological replicates. Different experiments are indicated by different data points in the violin plot (**e**). Error bars, s.d. Time course data in **d** and **f** were analyzed using two-way ANOVA with Fisher’s least significant difference test.

**Extended Data Fig. 4:**
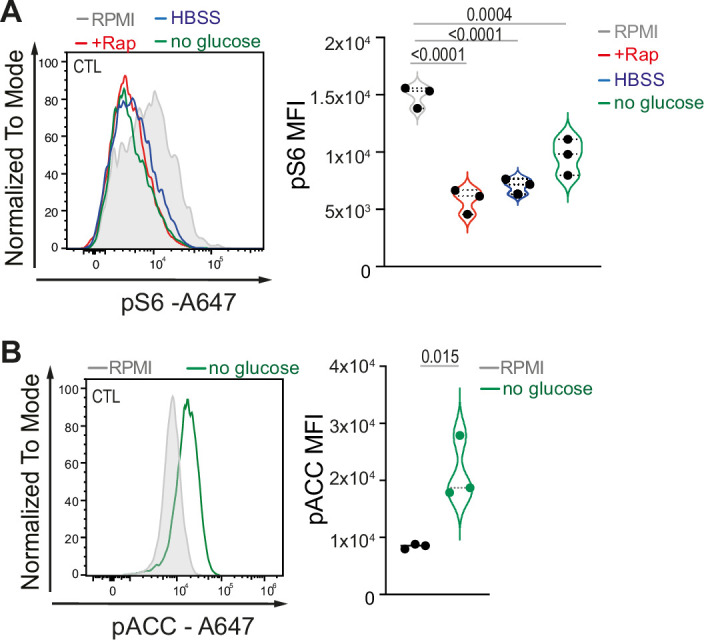
Nutrient regulation of mTORC1 and AMPK activity in CTL. Related to Fig. 4. **A** Flow cytometry histograms (left) and MFI (right) of ribosomal protein S6 phosphorylation (pS6) in CTL maintained in amino acid replete media (RPMI) with or without rapamycin (Rap, 20 nM), in amino acid depleted media (HBSS) or in glucose free RPMI (no glucose) for 18 h. **B** Flow cytometry histograms (left) and MFI (right) of ACC phosphorylation (pACC) in CTL maintained in regular, glucose containing media (RPMI) or in glucose free RPMI (no glucose) for 18hr. Data points in bar charts are indicative of biological replicates.

Phosphatidylinositol-3-kinase, vacuolar-protein-sorting-34 (VPS34) is critical for autophagosome formation^34–36^ and controls T cell autophagy^5,6,34,37^. In this context, high autophagy flux in amino-acid-deprived CTLs or in IL-7-maintained naive T cells is dependent on VPS34 activity^38^ (Fig. 4a,b). In some cell lineages, AMP-activated protein kinase (AMPK) can also control autophagy^39–41^ by repressing activation of mTORC1 (mammalian target of rapamycin complex 1)^39,40,42–44^. Therefore, we assessed whether AMPK activation or inhibition of mTORC1 controlled autophagy in CTL. The data show that amino acid deprivation or the mTORC1 inhibitor rapamycin suppresses mTORC1 activity (Extended Data Fig. 4a), but only amino acid deprivation, not rapamycin, induces CTL autophagy (Fig. 4c,d). Moreover, glucose deprivation, which activates AMPK and inhibits mTORC1^40^ (Extended Data Fig. 4a,b), does not induce autophagy (Fig. 4c,d). We also found no significant difference in basal levels of autophagy in wild-type and AMPK-null CTLs, and acute removal of amino acids but not glucose induced autophagy in both wild-type and AMPK-null CTLs (Fig. 4e). One other candidate autophagy regulator is the kinase GCN2 (ref. ^45–49^). Halofuginone is a GCN2 agonist that induces amino acid starvation responses in T cells^50–53^. The present data show that halofuginone induces autophagic flux in CTLs (Fig. 4f).

### VPS34 control of autophagy and protein degradation in CTL

**Fig. 5:**
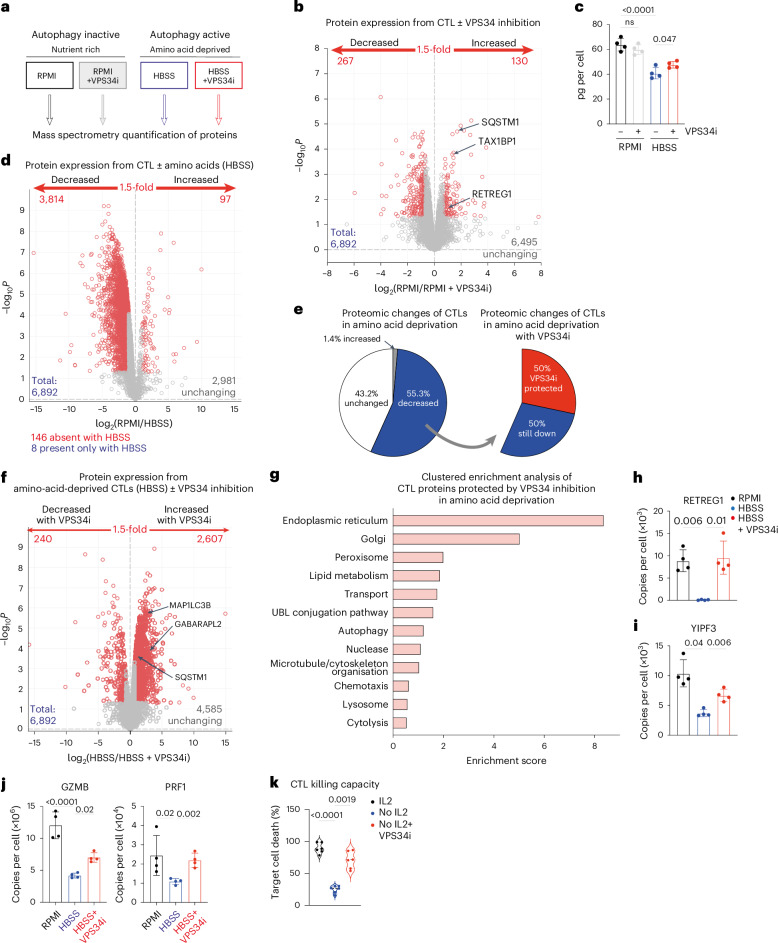
VPS34 controls autophagy/protein degradation in amino-acid-starved CTLs. **a**, Schematic diagram showing the four CTL treatment conditions (18 h) for subsequent proteomic analysis. **b**, Volcano plot showing ratio changes for proteins expressed in CTLs in RPMI with or without VPS34i treatment. *P* values below 0.05 and fold change above 1.5 are marked in red. **c**, Total protein content of CTLs in amino-acid-replete media (RPMI) or amino-acid-deprived media (HBSS) with or without VPS34i for 18 h. **d**, Volcano plot showing ratio changes for proteins in CTLs in RPMI compared with amino-acid-deprived (HBSS) CTLs. *P* values below 0.05 and fold change above 1.5 are marked in red. **e**, The proportion of the CTL proteome changed upon amino acid starvation (RPMI into HBSS; left) and the proportion of the amino-acid-starved CTL proteome that increased upon VPS34 inhibition (HBSS to VPS34i; right). **f**, Volcano plot showing ratio changes of proteins in amino-acid-deprived (HBSS) CTLs compared to amino-acid-deprived (HBSS) CTLs treated with VPS34i. *P* values below 0.05 and fold change above 1.5 are marked in red. **g**, Clustered enrichment analysis on proteins from amino-acid-deprived (HBSS) CTLs that were significantly increased with VPS34i treatment. UBL, ubiquitin and ubiquitin-like. **h**–**j** Mean protein copy numbers per cell of RETREG1 (**h**), YIPF3 (**i**) and GZMB and perforin (PRF1) (**j**). **k**, Target cell killing capacity of CTLs maintained in IL-2 or deprived of IL-2 with or without VPS34i for 24 h. Data are from *n* = 4 (**a**–**j**) and *n* = 6 (**k**) mice per condition. The full list of proteins and enrichment lists are available in Source Data Figs. 5, 6 and 7. Bar chart data indicate biological replicates. Error bars, s.d. Source data

The ability of VPS34 inhibitors to suppress autophagy in CTLs opens a strategy to explore how autophagy shapes CTL proteomes. Therefore, we used mass spectrometry to quantitively analyze CTL proteomes to identify proteins whose expression is VPS34-dependent in both nutrient-rich (low autophagy) or amino-acid-deprived (high autophagy) conditions (Fig. 5a). The rationale is that in CTLs undergoing autophagy, VPS34 inhibition of autophagy would increase the expression of proteins degraded by autophagy and decrease the expression of proteins synthesized using amino acids recycled by autophagy.

**Fig. 6:**
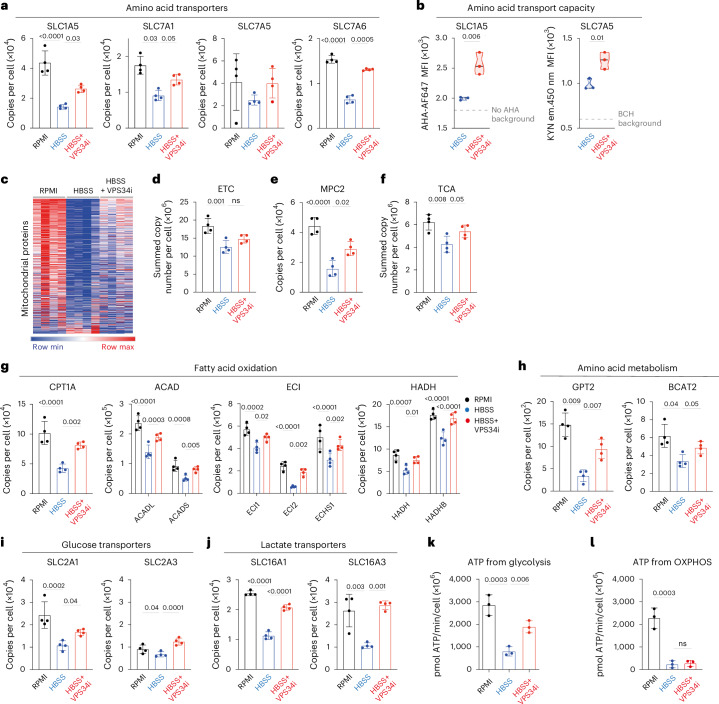
VPS34 controls metabolic remodeling in amino-acid-starved CTLs. **a**, Quantitative proteomics data showing mean protein copy numbers per cell of amino acid transporters SLC1A5, SLC7A1, SLC7A5 and SLC7A6 from CTLs in amino-acid-replete (RPMI) or amino-acid-deprived (HBSS) media with or without VPS34i for 18 h. **b**, SLC1A5 transport capacity as measured by azidohomoalanine (AHA) uptake (left) and SLC7A5 transport capacity as measured by KYN uptake (right) from CTLs in amino-acid-replete (RPMI) or amino-acid-deprived (HBSS) media with or without VPS34i for 18 h. **c**, Heatmap of mitochondrial proteins (Gene Ontology GO:0005739; MitoCarta3.0) from CTLs in amino-acid-replete (RPMI) or amino-acid-deprived (HBSS) media with or without VPS34i for 18 h. **d**, Summed protein copy number of electron transport chain (ETC; MitoCarta3.0 ‘OXPHOS’). **e**, Protein copies per cell of MPC2. **f**, Summed protein copy number of TCA cycle proteins (KEGG module M00009). **g**–**j**, Protein copy numbers per cell of enzymes and regulators involved in fatty acid oxidation (**g**), amino acid metabolism (**h**), glucose transport (**i**) and lactate transport (**j**) as indicated. **k**,**l**, Predicted ATP generated from glycolysis (**k**) or OXPHOS (**l**) by CTLs maintained in amino-acid-replete (RPMI) or amino-acid-deprived (HBSS) media with or without VPS34i for 18 h. Data are from *n* = 4 (**a**–**j**), *n* = 3 (**k**,**l**) mice per condition. The full list of proteins and enrichment lists are available in Source Data Figs. 5, 6 and 7. Bar chart and violin plots indicate biological replicates. Error bars, s.d. Source data

**Fig. 7:**
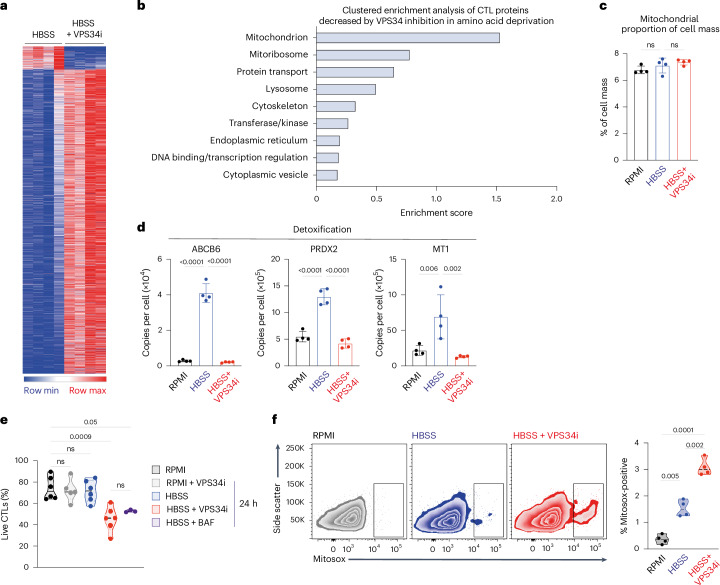
Survival programs in amino-acid-starved CTLs depend on VPS34. **a**, Heatmap of the subset of proteins from CTLs that are significantly changed upon amino acid deprivation (HBSS) and with VPS34i. **b**, Clustered enrichment analysis on proteins from amino-acid-deprived (HBSS) CTLs that were significantly decreased with VPS34i treatment. **c**, Protein content of mitochondrial proteins relative to total cell mass. **d**, Protein copy numbers per cell of ABCB6, PRXD2 and MT1 from CTLs maintained in amino-acid-replete (RPMI) or amino-acid-deprived (HBSS) media with or without VPS34i. **e**, Proportion live CTLs in amino-acid-replete (RPMI) or amino-acid-deprived (HBSS) media with or without VPS34i or BAF for 18 h. **f**, Representative flow cytometry data showing Mitosox staining (left) and proportion of Mitosox-high cells (right) from CTLs in amino-acid-replete (RPMI) or amino-acid-deprived (HBSS) media with or without VPS34i for 18 h. Data are from *n* = 4 (**a**–**d**), *n* = 6 (**e**) and *n* = 4 (**f**) mice per condition. The full list of proteins and enrichment lists are available in Source Data Figs. 5, 6 and 7. Bar chart and violin plots indicate biological replicates. Error bars, s.d. Source data

**Extended Data Fig. 5:**
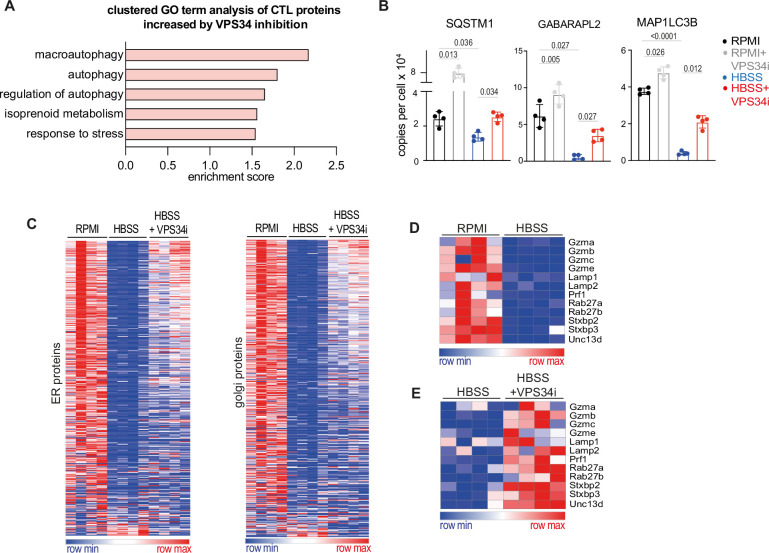
VPS34 control of autophagy/protein degradation in amino-acid-deprived CTLs. Related to Fig. 5. **A** Clustered enrichment analysis on proteins from amino acid replete (RPMI) CTL that were significantly increased with VPS34i treatment. The full enrichment table is available in Source Data Figs. 5, 6 and 7. **B** Quantitative proteomics data showing mean protein copy numbers per cell of SQSTM1, GABARAPL2 and MAP1LC3B. **C** Heatmap of ER proteins (GO:0005789) and Golgi proteins (GO:0005794) from CTL in replete amino acids (RPMI), or amino acid deprived (HBSS) with or without VPS34i for 18-hrs. **D** Heatmap of cytotoxicity related proteins from CTL in replete amino acids (RPMI), or amino acid deprived (HBSS) for 18-hrs. **E** Heatmap of cytotoxicity related proteins from amino acid deprived (HBSS) CTL +/- VPS34i for 18-hrs. Data points in bar charts are indicative of biological replicates. Error bars represent the mean +/- SD.

We first investigated the effect of VPS34 inhibition on CTLs cultured in amino-acid-replete medium (low autophagy flux). A total of 6,892 proteins were quantified but only 130 proteins increased expression following VPS34 inhibition (Fig. 5b and Source Data Figs. 5, 6 and 7). These proteins were predominately autophagy cargo carriers; for example, p62/SQSTM1, MAP1LC3B and GABARAPL2 (Fig. 5b, Extended Data Fig. 5a,b and Source Data Figs. 5, 6 and 7). The limited impact of VPS34 inhibition on CTL proteomes when cells are not undergoing autophagy highlights how VPS34-dependent pathways are not needed for IL-2 signaling or for the control of protein synthesis and cell metabolism under nutrient-replete conditions.

We then looked at the impact of VPS34 inhibition on proteomes of amino-acid-deprived CTLs (high autophagic flux). Amino acid deprivation (18 h) causes CTLs to decrease the expression of approximately 3,800 proteins and lose approximately one-third of their cell mass (Fig. 5c,d). Proteins downregulated under these conditions will include proteins degraded by the proteosome but then not resynthesized because of the unavailability of amino acids. There will also be proteins whose expression is decreased because of the high autophagy in amino-acid-deprived CTLs. These proteins can be identified by their failure to be degraded when VPS34 inhibitors are used to prevent autophagy. Here, the striking result was that VPS34 inhibition repressed the degradation of >2,000 proteins in amino-acid-deprived T cells; that is, approximately 50% of the proteins downregulated in amino-acid-deprived CTLs were degraded by autophagy (Fig. 5e,f). Cell mass losses in amino-acid-deprived CTLs were also blunted by autophagy inhibition (Fig. 5c).

Proteins ‘protected’ from degradation in amino-acid-deprived autophagy-inhibited CTLs included autophagy machinery SQSTM1, GABARAPL2 and MAP1LC3B (Fig. 5f and Extended Data Fig. 5b). One dominant effect of autophagy inhibition in amino-acid-deprived CTLs was the retained expression of endoplasmic reticulum and Golgi proteins and ER-phagy and Golgi-phagy receptors (Fig. 5g–i, Extended Data Fig. 5c and Source Data Figs. 5, 6 and 7). Enrichment analysis also highlighted that proteins controlling cytolytic functions were retained by autophagy inhibition (Fig. 5g and Extended Data Fig. 5d,e). Notably, amino-acid-deprived CTLs downregulated the expression of perforin and granzymes (Fig. 5j and Extended Data Fig. 5d) but the full loss of these cytolytic effector molecules was prevented by autophagy inhibition (Fig. 5j and Extended Data Fig. 5e). Furthermore, autophagic IL-2-deprived CTLs (Fig. 2a) have reduced cytotoxicity, but this is restored when VPS34 inhibitors are used to repress autophagy (Fig. 5k). This indicates that granzymes and perforin are degraded by autophagy, and autophagy repression sustains their expression to sustain the killing capacity of the CTLs. These observations offer a molecular explanation for improved tumor control by autophagy-deficient T cells^11,12^.

One other important category of proteins downregulated in amino-acid-starved T cells but protected by autophagy inhibition is amino acid transporters (Fig. 6a). The results predict that autophagy inhibition would increase the amino acid transport capacity of amino-acid-starved CTLs. We examined the amino acid transport capacity of CTLs that were starved of amino acids with and without autophagy inhibition using single-cell assays for the System L transporter SLC7A5 and the glutamine transporter SLC1A5 (refs. ^30,31^). Fig. 6b shows that the SLC1A5-mediated and SLC7A5-mediated transport of amino acids in starved CTLs is indeed increased upon VPS34i treatment.

**Extended Data Fig. 6:**
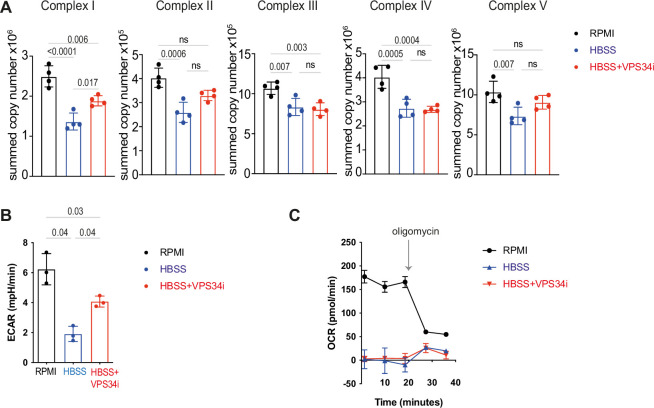
Amino-acid starvation induced mitochondrial remodelling and function. Related to Fig. 6. **A** Summed protein copy number of Complex I-V proteins of the mitochondrial electron transport chain (annotations: MitoCarta3.0 ‘OXPHOS’). **B** Basal ECAR of CTL maintained in amino acid replete media (RPMI) or amino acid deprived (HBSS) with or without VPS34i for 18-hrs. **C** Basal and oligomycin treated OCR measurements of CTL maintained in amino acid replete media (RPMI) or amino acid deprived (HBSS) with or without VPS34i for 18-hrs. Data points in bar charts are indicative of biological replicates. Error bars represent the mean +/- SD.

Finally, amino-acid-deprived autophagic CTLs showed selective mitochondrial remodeling (Fig. 6c). Some, but not all, of this remodeling was prevented by autophagy inhibition. Hence, mitochondrial proteins involved in electron transport chains were downregulated by amino acid starvation, but these losses were not prevented by autophagy inhibition (Fig. 6d and Extended Data Fig. 6a). By contrast, expression of the mitochondrial pyruvate carrier MPC2 (Fig. 6e), TCA cycle proteins (Fig. 6f), key proteins involved in fatty acid oxidation (Fig. 6g) and mitochondrial amino acid metabolism (Fig. 6h) were decreased upon amino acid deprivation but protected by autophagy inhibition. Repression of autophagy also increased glucose and lactate transporter levels in amino-acid-starved CTLs (Fig. 6i,j). Glucose transporters supply glucose to fuel ATP production from oxidative phosphorylation and glycolysis^54,55^. Lactate transporters are also rate-limiting for glycolysis^55^. These data predict that amino-acid-deprived T cells will downregulate glycolysis, but inhibition of autophagy will prevent this. The data also predict reductions in oxidative phosphorylation in amino-acid-deprived CTL, but the failure of autophagy inhibition to prevent the loss of major oxidative phosphorylation components (Fig. 6d and Extended Data Fig. 6a) predicts that these changes would not be controlled by autophagy. These predictions were tested using a Seahorse extracellular flux analyzer to assess the impact of autophagy induction and repression on rates of ATP generation from glycolysis and oxidative phosphorylation^56,57^. The data show decreased glycolysis and a corresponding calculated decrease in ATP production from glycolysis in amino-acid-deprived CTLs (Fig. 6k and Extended Data Fig. 6b). When autophagy is repressed in amino-acid-deprived CTLs, glycolysis and ATP production from glycolysis increases (Fig. 6k and Extended Data Fig. 6b). There is also decreased oxygen consumption by amino-acid-deprived CTLs, with a major impact on calculated ATP production from oxidative phosphorylation; these changes were not rescued by autophagy inhibition (Fig. 6l and Extended Data Fig. 6c).

### CTL survival is orchestrated by autophagy during amino acid starvation

**Extended Data Fig. 7:**
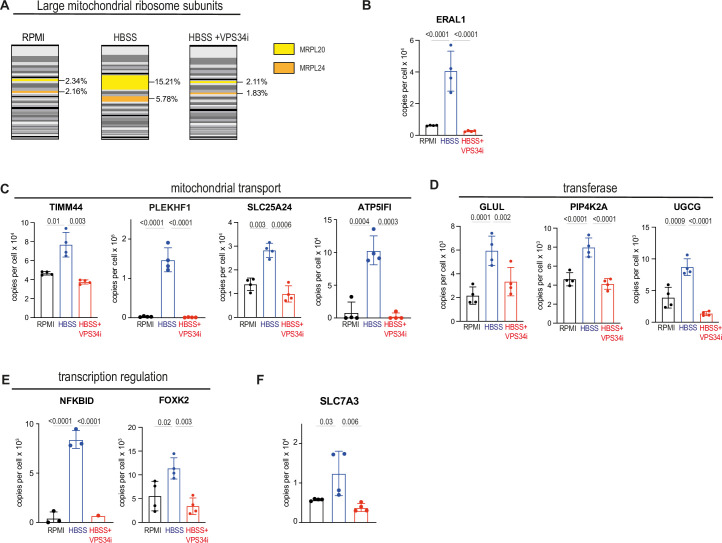
VPS34-dependent proteome remodelling in response to amino-acid starvation. Related to Fig. 7. **A** MRPL20 and MRPL24 expression as a percentage of all large mitochondrial ribosome subunits. **B-F** Protein copy numbers per cell of ERAL1 (B); proteins involved in mitochondrial transport (C); transferases (D); transcriptional regulation (E) and SLC7A3 (F). Data points in bar charts are indicative of biological replicates. Error bars represent the mean +/- SD.

Proteins fueled by autophagy-recycled amino acids can be identified as proteins whose abundance decreased with autophagy inhibition in amino-acid-deprived CTLs (Fig. 7a and Source Data Figs. 5, 6 and 7). These include mitochondrial proteins, protein transport, lysosomal and vesicle proteins as well as DNA binding proteins (Fig. 7b and Source Data Figs. 5, 6 and 7). There was no increase in relative mitochondrial mass upon amino acid deprivation (Fig. 7c); instead, amino acid deprivation drives mitochondrial remodeling with the expression of mitoribosomes, mitoribosome assembly factors and mitochondrial transporters dependent on autophagy (Extended Data Fig. 7a–c). Other proteins dependent upon autophagy for expression include metabolic enzymes, NFKB inhibitory proteins and the arginine transporter SLC7A3 (Extended Data Fig. 7d–f); the latter is known to be upregulated by amino acid deprivation^58^. Autophagy in amino-acid-starved CTLs also fueled the expression of proteins associated with cell detoxification. These include ABCB6, which is crucial for heme biosynthesis and regulates catalase to protect cells from reactive oxygen species^59^; PRDX2, a peroxiredoxin that reduces hydrogen peroxide and alkyl hydroperoxides and has an antioxidant protective role^60^; and MT1, a metallothionein previously shown to control dysfunctional T cells in anti-tumor responses^61,62^ (Fig. 7d). The autophagy-driven expression of a suite of ‘protective’ proteins raises questions about whether inhibiting autophagy in amino-acid-deprived CTLs impacts cell survival. Here, a salient result is that the inhibition of autophagy has no impact on cell survival in amino-acid-replete CTLs, whereas amino-acid-deprived CTLs accumulate high levels of intracellular reactive oxygen species and die when autophagy is blocked (Fig. 7e,f).

### VPS34 control of autophagy and protein degradation in naive CD8 T cells

**Fig. 8:**
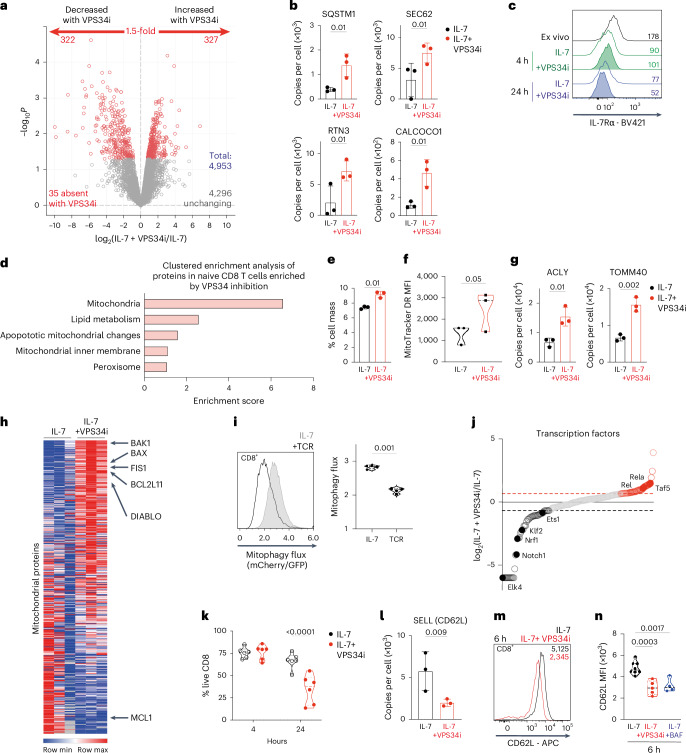
VPS34 control of autophagy and protein degradation in naive CD8 T cells. **a**, Volcano plot showing ratio changes of proteins in IL-7-maintained CD8 T cells treated with VPS34i for 5 h. *P* values below 0.05 and fold change above 1.5 are marked in red. **b**, Protein copies per cell of SQSTM1, SEC62, RTN3 and CALCOCO1. **c**, Flow cytometry staining of IL-7Rα expression on CD8 T cells, either ex vivo or maintained in IL-7 with or without VPS34i for 4–24 h. MFI values are presented. **d**, Clustered enrichment analysis of proteins increased in naive CD8 T cells treated with VPS34i for 5 h **e**–**f**, Mitochondrial protein content relative to total cell mass (**e**) and fluorescence intensity (MFI) values of MitoTracker Deep Red staining (**f**) of CD8 T cells maintained in IL-7 with or without VPS34i for 5 h. **g**, Protein copies per cell of ACLY and TOMM40 from IL-7-maintained CD8 T cells ± VPS34i for 5 h. **h**, Heatmap of mitochondrial proteins (GO:0005739; MitoCarta3.0). **i**, Mitophagy flux profiles of mitophagy reporter CD8 cells maintained in IL-7 or activated through the TCR (aCD3/CD28) for 6 h (left) and corresponding mean mitophagy flux values (right). **j**, Ranked expression of transcription factors (GO:00037000) detected by proteomics. A 1.5-fold decrease with VPS34i is shown in black, and a 1.5-fold increase with VPS34i is shown in red. **k**, Percentage live CD8 T cells maintained in IL-7 with or without VPS34i for 4–24 h. **l**, Protein copies per cell of SELL (CD62L) from CD8 T cells maintained in IL-7 ± VPS34i for 5 h. **m**, CD62L surface expression on CD8 T cells maintained in IL-7 with or without VPS34i for 6 h; corresponding MFI values are presented in the plot. **n**, Flow cytometry MFI values of CD62L surface staining from CD8 T cells maintained in IL-7 ± VPS34i or BAF for 6 h. Data are from *n* = 3 (**a**–**j**,**l**), *n* = 6 (**k**) and *n* = 4–8 (**m**,**n**) mice per condition. The full list of proteins and enrichment lists are available in Source Data Fig. 8. Bar chart and violin plots indicate biological replicates. Error bars, s.d. Source data

Naive T cells are dependent on autophagy^1,5^; to understand why, we used mass spectrometry to quantitatively analyze how autophagy shapes naive T cell proteomes. In these experiments, proteomes of IL-7-maintained naive T cells cultured in the presence or absence of the VPS34 (autophagy) inhibitor for 5 h were compared. More than 4,900 proteins were quantified; autophagy inhibition increased the abundance of 327 proteins and decreased the expression of ~300 (Fig. 8a and Source Data Fig. 8). Autophagy flux repression was confirmed by the increased expression of autophagy cargo adaptors p62/SQSTM1, SEC62, RTN3 and CALCOCO1 in VPS34i-treated cells (Fig. 8b). Previous studies^19^ have identified IL-7 receptors as autophagy cargo in proliferating CD4 T cells^19^. However, autophagy inhibition did not cause the accumulation or prevent the internalization of IL-7R in naive CD8 T cells (Fig. 8c).

**Extended Data Fig. 8:**
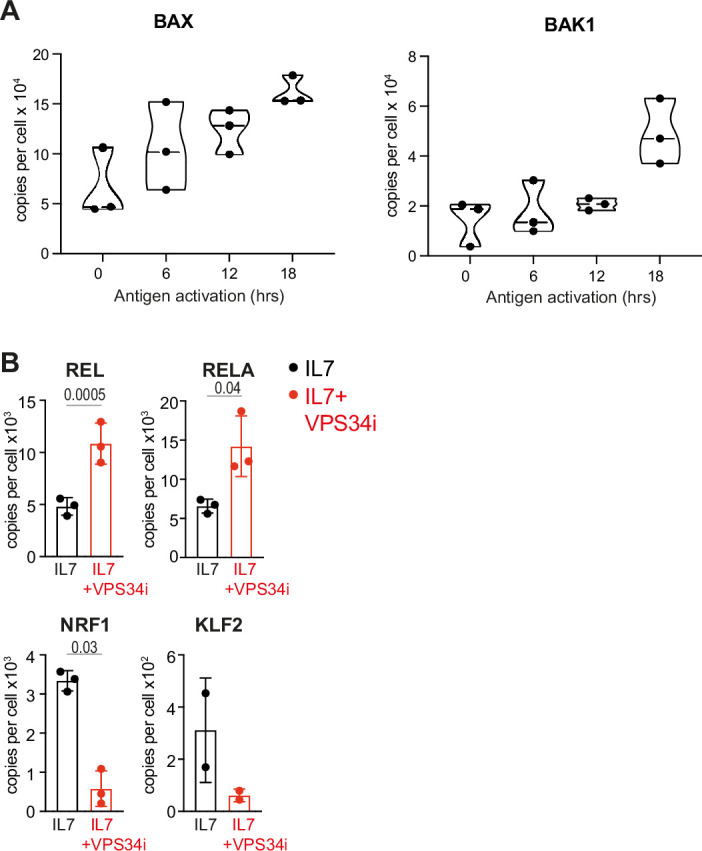
Regulation of protein expression in CD8 T cells. Related to Fig. 8. **A** Mean protein copy numbers of BAX and BAK1 from naïve and OT1 CD8 T cells activated with SIINFEKL for the indicated times. Data available on Immpres.co.uk^22^. **B** Mean protein copy numbers of REL, RELA, NRF1 and KLF2 from CD8 T cells maintained in IL-7 with or without VPS34i. Data points in bar charts or violin plots are indicative of biological replicates. Error bars represent the mean +/- SD.

Clustered enrichment analysis suggested that the dominant effect of autophagy inhibition in naive T cells was the accumulation of mitochondrial proteins (Fig. 8d and Source Data Fig. 8), and Fig. 8e,f shows increased mitochondrial mass in autophagy-inhibited naive T cells. Structural proteins, including membrane transporters, mitochondrial ribosomal subunits and mitochondrial enzymes, all accumulate in response to autophagy inhibition (Fig. 8g and Source Data Fig. 8). This is consistent with previous studies showing that VPS34-null T cells increase mitochondrial mass^5^. The insight from the present study is that the increased mitochondrial mass that occurs following autophagy inhibition is not scaling up of normal mitochondria but rather reflects the accumulation of abnormal mitochondria (Fig. 8h). Notably, VPS34-inhibitor-treated naive CD8 T cells accumulate the pro-apoptotic mitochondrial pore proteins BAK1, BIM (BCL2L11), DIABLO and BAX and the mitochondrial fission-related protein FIS1 (Fig. 8h). In this context, the suppression of autophagy flux in naive T cells following antigen receptor engagement is correlated with accumulation of BAK1 and BAX (Extended Data Fig. 8a). Hence, the dominant form of autophagy in naive T cells appears to be mitophagy, and T cell receptor (TCR)-induced suppression of autophagy flux in naive CD8 T cells is actually TCR-suppression of mitophagy. To test this idea, we used T cells expressing the mito-QC mitophagy reporter^23,24,63^ in which mCherry–GFP, fused to the mitochondrial targeting sequence FIS1, localizes to outer mitochondrial membranes. GFP fluorescence is quenched when the reporter is recruited to autophagosomes. Fig. 8i shows GFP/mCherry fluorescence profiles of the mitophagy reporter and shows that naive CD8 T cells have high mitophagy flux, which is decreased upon TCR activation.

We next wondered what proteins are fueled by amino acids that are recycled by autophagy in naive T cells. Here, candidates are proteins whose abundance was decreased by autophagy inhibition (Fig. 8a and Source Data Fig. 8). Notably, autophagy inhibition caused decreased expression of KLF2, ETS family and Nrf1 transcription factors (Fig. 8j and Extended Data Fig. 8b) and the anti-apoptotic family member MCL-1 (Fig. 8h). MCL-1 mediates pro-survival responses of IL-7 in naive T cells. Thus, loss of MCL-1 predicts that autophagy inhibition in naive CD8 T cells would impact cell survival. Fig. 8k shows that VPS34 inhibition of IL-7-maintained naive CD8 T cells for 24 h results in increased death (Fig. 8k). One other salient observation was that VPS34 inhibition decreased the abundance of l-selectin (CD62L), a critical adhesion receptor that controls naive T cell trafficking into secondary lymphoid tissues (Fig. 8l)^64–66^. This observation was tested orthogonally using flow cytometry, which also showed decreased expression of CD62L in VPS34-inhibitor-treated naive CD8 cells (Fig. 8m,n). CD62L has a short half-life in naive T cells and needs to be constantly synthesized to maintain membrane expression^67^. The present data reveal that CD62L re-synthesis in naive cells uses amino acids from proteins that have been degraded by autophagy.

## Discussion

The present study improves our understanding of autophagy regulation during T cell immune responses. Fundamental discoveries are that autophagy, particularly mitophagy, is high in naive T cells and that immune activation acutely represses mitophagy and autophagy flux and represses protein degradation by autophagy. T cell autophagy flux is controlled by amino acid sensing; however, a unique dimension to autophagy control in T cells is that the ability of T cells to ‘sense’ amino acids is regulated by antigen receptor and cytokine control of amino acid transporter expression. The default state of high autophagy in naive or memory T cells reflects that these have low amino acid transporter expression and a low ability to sense amino acid environments. Autophagy repression occurs when immune-activated T cells upregulate amino acid transporter expression. High levels of amino acid transport capacity in activated T cells allow autophagy repression when cells are in amino-acid-replete environments. Immune-activated cells rapidly revert to high autophagy flux when amino acids are deprived. The sustained expression of amino acid transporters by T cells is dependent on sustained stimulation by antigen receptors or pro-inflammatory cytokines^32,33,68^. Accordingly, the transient nature of autophagy repression in T cells responding to immune activation reflects the restriction of amino acid transporter expression to T cells acutely responding to antigens or cytokines. Once antigen and inflammatory cytokine levels decline, amino acid transporter expression declines and cells revert to high autophagy flux. One key observation here is that not all cytokines are equal autophagy regulators. For example, IL-2, IL-12 and IL-18, which drive CD8 effector differentiation, repress autophagy, whereas IL-15 and IL-7, which support naive and memory T cells, do not. This differential ability of cytokines to repress autophagy thus reflects their different abilities to control amino acid transporter expression.

Many previous studies have observed increased levels of autophagosomes in effector T cells and concluded that immune activation of T cells increases autophagy^1,2,13–18,23^. Our data confirm that the expression of autophagic machinery is highly increased in immune-activated cells. However, that increased expression of the autophagy machinery does not mean cells increase autophagy flux. Indeed, autophagosomes accumulate in activated T cells because autophagy flux repression by antigens and inflammatory cytokines prevents autophagy machinery degradation^7^. Autophagy flux is thus at its highest in naive and memory T cells, which fits with genetic studies showing that the loss of autophagy regulators causes loss of these populations^1–9^. The question becomes what the significance is of autophagy repression by antigens and inflammatory cytokines. Here, understanding is helped by identifying proteins and organelles that are degraded and fueled by autophagy. One key insight is that there are fundamental differences in autophagy cargoes in naive versus effector CD8 T cells. Dominant cargoes for autophagy in naive T cells are mitochondrial proteins. By contrast, in nutrient-deprived cytotoxic T cells, mitochondria are not the dominant proteins degraded by autophagy; indeed the opposite, as amino acids produced by autophagy fuel mitochondrial remodeling. Rather, dominant autophagy substrates in effector T cells are nutrient transporters, glycolytic enzymes and cytotoxic granule contents. Effector T cells thus use autophagy to downregulate energy-consuming processes to fuel proteins that control energy-conserving pathways. Naive T cells use autophagy to prune damaged mitochondria to maintain mitochondrial integrity and use amino acids recycled by autophagy to fuel the expression of molecules that are essential for naive T cell homeostasis; for example, CD62L, a key T cell homing receptor^1,64,65,67,69,70^. The biological ‘cost’ of autophagy repression is that it prevents cells from removing damaged organelles or unfolded proteins; the biological advantage is that repression of an energetically demanding protein degradation process allows cells to divert energy to support de novo synthesis of proteins that support T cell clonal expansion and differentiation rather than using ATP to replenish degraded proteins.

Finally, the importance of regulated expression of amino acid transporters for T cell immune responses has been well documented^29,32,71^. However, the present work reveals that immune regulation of amino acid transport capacity does not simply provide amino acids for de novo protein synthesis but is also a key autophagy checkpoint that affords a molecular mechanism for antigen receptors and inflammatory cytokines to repress autophagy flux, one of the major protein degradation pathways in T cells.

## Material and Methods

### Mice and cells

Mice used in this study included OT-1 TCR transgenic mice (Charles River; stock no. 003831)^75^; AMPKα1^fl/fl^ and CD4CreAMPKα1^fl/fl^ (ref. ^40^); C57BL/6 (wild-type; Charles River); autophagy reporter mice in which an mCherry–GFP–Map1lc3b (mCherry–GFP–LC3b) fusion protein is expressed ubiquitously from the *ROSA26* locus^23^; and mitophagy reporter (mito-QC) mice, in which an mCherry–GFP–mtFIS1 fusion protein is expressed ubiquitously from the *ROSA26* locus^23,24,63^. All mice were used at 8–12 weeks of age. Mice were group-housed according to litter size, with food and water available ad libitum, on a 12:12 h light-to-dark schedule and maintained at constant temperature (20 ± 1 °C) and humidity. All mice were maintained in the University of Dundee Biological Resource Unit in compliance with UK Home Office Animals (Scientific Procedures) Act 1986 guidelines.

T cells were cultured in RPMI 1640 medium containing glutamine (Gibco), supplemented with 10% FBS (Gibco), 1% penicillin–streptomycin (Gibco) and 50 µM β-mercaptoethanol (Sigma-Aldrich) (RPMI 1640 complete medium) at 37 °C and 5% CO_2._ Single-cell suspensions were generated by disaggregation of lymph nodes or spleens. Splenic red blood cells were lysed in ACK buffer (150 mM NH_4_Cl, 10 mM KHCO_3_, 110 µM Na_2_EDTA, pH 7.8). Cell suspensions were washed in complete culture medium before activation. To maintain naive T cells, splenic and lymph node single-cell suspensions were cultured with IL-7 (5 ng ml^−1^; PeproTech) in complete RPMI 1640 medium. VPS34-IN1 (ref. ^38^) (VPS34i; 1 µM), halofuginone (100 nM), bafilomycin A1 (200 nM), ruxolitinib (1 μM) and rapamycin (20 nM) were used where indicated. The EasySep Mouse CD8^+^ T Cell Isolation Kit (STEM Cell Technologies) was used to purify naive OT-1 CD8^+^ T cells for proteomic analysis.

### Polyclonal T cell activation

To generate CTLs, cells were stimulated with 1 μg ml^−1^ of CD3 antibody (2C11; BioLegend) and 2 μg ml^−1^ CD28 antibody (37.51; eBiosciences) in RPMI 1640 complete media for 48 h. Cells were then washed out of activation conditions and polyclonally expanded in RPMI 1640 complete media supplemented with IL-2 (20 ng ml^−1^, Proleukin; Novartis) at a density of 3 × 10^5^ cells per ml for a further 3–5 days.

### OT-I TCR transgenic T cell activation

For the generation of OT-1 CTLs, lymph node single-cell suspensions were isolated from OT-1 TCR transgenic mice and stimulated for 36 h with SIINFEKL peptide (10 ng ml^−1^). Cells were washed out of activation conditions and clonally expanded in RPMI 1640 complete media supplemented with IL-2 (20 ng ml^−1^, Proleukin; Novartis) at a density of 3 × 10^5^ cells per ml for a further 3–5 days.

For experiments with IL-12 and IL-18, activated OT-1 T cells were initially expanded in IL-2 (20 ng ml^−1^) and IL-12 (20 ng ml^−1^; Peprotech) before being switched into IL-2 alone, IL-12 + IL-18 (20 ng ml^−1^; R&D Systems) or no cytokine for 24 h. For experiments with IL-15, 36 h SIINFEKL-activated OT-1 T cells were expanded in IL-15 (20 ng ml^−1^; Peprotech).

### Nutrient-deprivation studies

Before nutrient deprivation, cells were washed twice in PBS. Cells were then resuspended at 3 × 10^5^ cells per ml in either complete RPMI or nutrient-deprivation medium listed below. All nutrient-deprivation media were supplemented with 10% dialyzed FBS (Gibco), 1% penicillin–streptomycin (Gibco) and 50 µM β-mercaptoethanol (Sigma-Aldrich).

- For total amino acid starvation, cells were cultured in Hanks’ Balanced Salt solution (HBSS, Gibco).
- For glucose starvation, cells were cultured in RPMI 1640 media lacking glucose (Gibco).
- For glutamine starvation, cells were cultured in RPMI 1640 media lacking glutamine (Gibco).
- For arginine or methionine starvation, cells were cultured in RPMI 1640 media lacking arginine, lysine and methionine (DC Biosciences). Supplementation of individual amino acids (Sigma-Aldrich) to the levels of standard RPMI 1640 formula was carried out to generate culture medium lacking a single amino acid, methionine or arginine.

### Flow cytometry sample acquisition and analysis

For surface staining, antibody clones used were CD8a (53-6.7), CD44 (IM7), IL-7Rα (A7R34), CD62L (MEL-14), Fc Block (BD Biosciences; cat. no. 55314; 1 µg per million cells). Antibodies conjugated to APC, AlexaFluor 647, PeCy7, PerCPCy5.5, APC-efluor780, Brilliant Violet 421 and 605 were obtained from either eBioscience, BioLegend or BD Biosciences. For cell surface staining, a 1:200 dilution of staining antibodies was used. For experiments with live–dead determination, DAPI (4′,6-diamidino-2-phenylindole; Thermo) was used as a viability indicator and added at 250 ng ml^−1^ before acquisition.

For intracellular staining, cells were fixed with 1% formaldehyde (v/v) before permeabilization with 90% (v/v) ice-cold methanol for 30 min. Cells were washed and incubated with antibody against phospho-S6 Ser235/236 (cat. no. 2211; 1:100) or phospho-ACC S79 (clone D7D11; cat no. 11818; 1:100) before incubation with anti-rabbit Alexa 647 secondary (cat no. 4414; 1:100; all Cell Signaling technology). Cells were washed and resuspended in 0.5% FBS/PBS (v/v) for flow cytometric acquisition.

Mitotracker Deep Red (cat. no. M22426) and MitoSOX (cat. no. M36008) staining was performed according to the manufacturer’s guidelines (Invitrogen, Thermo Scientific).

Kynurenine uptake to monitor System L transporter activity was done according to established protocols^31^. In brief, surface-stained cells were incubated in pre-warmed HBSS (Gibco) with 200 µM kynurenine (Sigma-Aldrich) for 4 min at 37 °C before fixation with 1% formaldehyde (v/v). When indicated, kynurenine uptake was performed with 10 mM BCH (Sigma-Aldrich), a competitive inhibitor for System L uptake. Cells were washed post fixation and resuspended in 0.5% FBS/PBS for flow cytometric acquisition. Kynurenine uptake was detected by emission at 450 nm.

SLC1A5-mediated uptake capacity was measured by using azide-substrate and copper-click labeled to a fluorophore for detection as previously described^30^. In brief, cells were incubated in pre-warmed HBSS (Gibco) with 100 uM azidohomoalanine (AHA; Cambridge Research) for 4 min at 37 °C before fixation with 1% formaldehyde (v/v). Cells were permeabilized with 0.01% saponin (Sigma-Aldrich) in PBS for 20 min before incubation with the click-mixture containing AZDye 647 Alkyne (Click Chemistry tools).

### Flow cytometric measurements of autophagy and mitophagy flux

An autophagy flux reporter comprising an mCherry–GFP–Map1lc3b (mCherry–GFP–LC3b) fusion protein expressed ubiquitously from the *ROSA26* locus was used. Autophagic flux can be quantified by analyzing GFP fluorescence quenching normalized to mCherry signals. Autophagy flux is measured by calculating the mCherry/GFP fluorescence ratio as a ‘derived parameter’ in FlowJo. mCherry/GFP ratio values of 1 indicate low autophagy; mCherry/GFP ratio values above 1 indicate high autophagy flux.

A mitophagy flux reporter comprising an mCherry–GFP–mtFIS1 fusion protein expressed ubiquitously from the *ROSA26* locus was also used^23,24^. Mitophagy flux is measured by calculating the mCherry/GFP fluorescence ratio as a ‘derived parameter’ in FlowJo.

Flow cytometry was performed on Novocyte (Agilent), FACSVerse or LSRFortessa (BD Biosciences) flow cytometers. During acquisition, lymphocytes were identified by gating on forward-scatter area (FSC-A) and side-scatter area (SSC-A). Doublets were excluded using FSC-A versus forward-scatter width (FSC-W). Specific experimental gating strategies are referred to in the figure legends. FlowJo analysis software (v.10.9 and v.10.10; Becton Dickinson) was used to analyze all flow cytometry experiments.

### *Listeria* monocytogenes infection model

Mice were intravenously infected with 1–2 × 10^6^ colony forming units of attenuated ActA-deficient *L. monocytogenes*^76^. On days 1, 3 and 7 post infection, spleens were dissected and analyzed by flow cytometry.

### MC38 cell line culture and MC38 tumor model

The MC38 colon cancer cell line was purchased from Kerafast. MC38 cells were grown in DMEM (Gibco) supplemented with 10% FBS (Gibco), 2 mM glutamine, 0.1 mM non-essential amino acids, 1 mM sodium pyruvate, 10 mM HEPES, 50 μg ml^−1^ gentamycin sulfate and 1% penicillin–streptomycin. Cells were split 1:10 every 2–3 days when cells were ~80% confluent using 0.05% trypsin-EDTA (Gibco). MC38 cells were passaged two or three times before preparation for injection. On the day of injection, cells were prepared at 2 × 10^5^ cells per ml in RPMI 1640, and 3 × 10^4^ cells were injected into the right, shaved flank of each mouse. Tumor height and width were measured daily using electronic calipers from 3 days post injection onwards. Mice were killed on a predetermined day (day 12), or earlier if a welfare issue occurred.

Tumor digestion was performed using the magnetic activated cell sorting (MACS) Biotec Mouse Tumor Dissociation Kit as per the manufacturer’s protocol (Miltenyi; cat. no. 130-096-730). Tumors were cut into 2–4 mm pieces and transferred to a gentleMACS column (Miltenyi), followed by the addition of 2.625 ml of a digestive enzyme cocktail (Miltenyi). The tube was inverted and placed onto the gentleMACS Dissociator (Miltenyi). The 37_m_TDK_1 program for soft/medium tumor digestion was used to generate a single-cell suspension that could be analyzed by flow cytometry.

### CTL killing assay

OT-1 CTLs were maintained in IL-2 (20 ng ml^−1^) or deprived of IL-2 with or without VPS34i (1 µM) for 24 h before co-culture with E.G7-OVA (ATCC, CRL-2113) target cells in a 5:1 target to T cell ratio. E.G7-OVA target cells were stained using CellTracker Deep Red (ThermoFisher) according to the manufacturer’s instructions 24 h before the killing assay. The killing capacity of the CTLs is represented by the percent of target cells undergoing apoptosis after 4 h. The co-culture was stained with CellEvent-Caspase3/7-Green (ThermoFisher) for 30 min, then fixed in IC Fixation Buffer (Invitrogen), left for 30 min at room temperature (19–21 °C), then washed twice in PBS and analyzed with an iQue Screener (Sartorius). CellTracker-stained cells with high-intensity CellEvent-Caspase3/7-Green were determined to be apoptotic target cells.

### Metabolic assays

The oxygen consumption rate and extracellular acidification rate of CTLs, either maintained with amino acids (complete RPMI) or starved of amino acids for 18 h (HBSS) ± VPS34i, were measured using a Seahorse XF24 analyzer and following established protocols^41,56,77^. In brief, 2 × 10^5^ CTLs were plated in poly-d-lysine-coated XF24 plates and gently centrifuged in appropriate XF medium. Data were normalized to cell number, and basal and oligomycin-treated measurements were used to determine ATP production by OXPHOS or glycolysis as previously described^57^.

### Proteomics sample preparation

Cell pellets were prepared for mass spectrometry analysis as described previously^78^. In brief, cells were lysed in 400 μl lysis buffer (5% SDS, 10 mM tris(2-carboxyethyl)phosphine, 50 mM triethylammonium bicarbonate) and incubated for 5 min at room temperature, shaking at 1,000 rpm. Samples were then incubated at 95 °C for 5 min at 500 rpm and then shaken again at room temperature as described above. Samples were sonicated for 15 cycles of 30 s on and 30 s off with a BioRuptor (Diagenode). To remove trace DNA contamination, 1 μl benzonase was added to each sample followed by incubation at 37 °C for 15 min. Protein concentration was determined using the EZQ quantification kit (Thermo Fisher Scientific). Samples were then alkylated with the addition of iodoacetamide to a final concentration of 20 mM and incubated for 1 h in the dark at 22 °C. Protein lysates were processed using S-Trap mini columns (Protifi) following the manufacturer’s instructions. Eluted peptides were dried overnight before being resuspended in 50 µl 1% formic acid, and peptide concentration was measured using the CBQCA assay following the manufacturer’s protocol (Thermo Fisher Scientific).

### Mass spectrometry

Peptides generated from IL-7-maintained naive cells and CTLs were analyzed by data-independent acquisition (DIA) on a Q Exactive HF-X (Thermo Scientific) mass spectrometer coupled with a Dionex Ultimate 3000 RS system (Thermo Scientific). A total of 1.5 µg of peptide from each sample was analyzed as described previously^79^. Liquid chromatography buffers were as follows: buffer A (0.1% formic acid in Milli-Q water (v/v)) and buffer B (80% acetonitrile and 0.1% formic acid in Milli-Q water (v/v)). A 1.5 μg aliquot of each sample was loaded at 15 μl min^−1^ onto a trap column (100 μm × 2 cm, PepMap nanoViper C18 column, 5 μm, 100 Å; Thermo Scientific) equilibrated in 0.1% trifluoroacetic acid. The trap column was washed for 3 min at the same flow rate with 0.1% trifluoroacetic acid and then switched in-line with a resolving C18 column (75 μm × 50 cm, PepMap RSLC C18 column, 2 μm, 100 Å; Thermo Scientific). The peptides were eluted from the column at a constant flow rate of 300 nl min^−1^ with a linear gradient from 3% buffer B to 6% buffer B in 5 min, then from 6% buffer B to 35% buffer B in 115 min and finally to 80% buffer B within 7 min. The column was then washed with 80% buffer B for 4 min and re-equilibrated in 3% buffer B for 15 min. Two blanks were run between each sample to reduce carry-over. The column was kept at a constant temperature of 50 °C at all times.

The data were acquired using an easy spray source operated in positive mode with spray voltage at 1.9 kV, capillary temperature at 250 °C and funnel RF at 60 °C. The mass spectrometer was operated in DIA mode. A scan cycle comprised a full mass spectrometry scan (*m*/*z* range from 350 to 1,650, with a maximum ion injection time of 20 ms, a resolution of 120,000 and an automatic gain control value of 5 × 106). The mass spectrometry survey scan was followed by tandem mass spectrometry DIA scan events using the following parameters: default charge state of three, resolution 30.000, maximum ion injection time 55 ms, automatic gain control of 3 × 106, stepped normalized collision energy 25.5, 27 and 30, and fixed first mass 200 *m*/*z*. Data for both mass spectrometry and tandem mass spectrometry scans were acquired in profile mode. Mass accuracy was checked before the start of sample analysis.

### Mass spectrometry data analysis

Raw DIA mass spectrometry data files were searched using Spectronaut (v.16.0.220606.53000; Biognosys). Data were analyzed using a hybrid library approach. For IL-7-maintained cells, the library was assembled using a deep proteome of naive CD8 T cells along with the experimental DIA samples. For CTLs, the library was assembled using a deep proteome of CTLs along with experimental DIA samples. Naive and CTL deep proteomes were generated by fractionating peptides from these samples using high-pH reverse-phase fractionation and analyzing by data-dependent acquisition. A 1 µg sample of peptide from each fraction was analyzed using an LTQ Orbitrap Velos (Thermo Fisher Scientific) as described in detail previously^71^. Raw mass spectrometry library data files were searched using the Pulsar tool within Spectronaut, using the following settings and as described previously^78^: a 0.01 false discovery rate at the protein and peptide level with digest rule set to ‘TrypsinP’. A maximum of two missed cleavages and a minimum peptide length of seven amino acids was selected. Carbamidomethyl of cysteine was selected as a fixed modification while protein amino-terminal acetylation and methionine oxidation were selected as variable modifications. The data were searched against a mouse database from UniProt (release June 2020). The database was generated using all manually annotated mouse SwissProt entries, combined with mouse TrEMBL entries with protein-level evidence available and a manually annotated homolog within the human SwissProt database. Experimental DIA samples were then searched against both the fractionated data-dependent acquisition library and the experimental DIA library using the following identification settings: protein and precursor *q*-value set to 0.01 with an ‘inverse’ decoy method and ‘dynamic’ decoy limit strategy. The following quantification settings were used: ‘Quant 2.0’; the mass-spectrometry-level quantity was set to ‘MS2’; imputation was disabled; major group Top N and minor group Top N were set as ‘false’; and cross-run normalization was set as ‘false’.

Protein copy numbers per cell and protein abundances were estimated using the ‘proteomic ruler’^80^. *P* values and fold changes for volcano plots were calculated using RStudio (v.2023.06.1+524) with the Bioconductor package ‘limma’ (v.3.54.2)^81^.

Heatmaps were generated using the Morpheus data visualization tool hosted by the Broad Institute (Morpheus; https://software.broadinstitute.org/morpheus).

### Statistical analysis

Data are presented as mean values ± s.d. unless otherwise stated. Exact *P* values are indicated in the figures. Statistical analyses were performed using Prism 10 (GraphPad Software). The Shapiro–Wilk test for normality and Brown–Forsythe test for heterogeneity of variance were used to determine suitability for parametric testing. An unpaired two-tailed *t*-test was used to compare different media or activation or cytokine conditions. A paired samples *t*-test was used to compare treatment with or without inhibitor. Multiple comparisons in one-way ANOVA analyses were corrected using the Tukey method. Alternative tests used are stated in the respective figure legends.

Gene Ontology term analysis and Kyoto Encyclopedia of Genes and Genomes pathway analysis were completed by using the online software tool DAVID (v.2023q4)^82,83^.

### Reporting summary

Further information on research design is available in the Nature Portfolio Reporting Summary linked to this article.

## Online content

Any methods, additional references, Nature Portfolio reporting summaries, source data, extended data, supplementary information, acknowledgements, peer review information; details of author contributions and competing interests; and statements of data and code availability are available at 10.1038/s41590-025-02090-1.

## Supplementary information

Reporting Summary

## Source data

Source Data Fig. 1 Source data for Fig. 1b,c. Data are from www.Immpres.co.uk

Source Data Figs. 5, 6 and 7 Source proteomics data for Figs. 5, 6 and 7: Proteomics data (copies per cell) from CTLs in RPMI (replete amino acids) ± VPS34i, and HBSS (amino acid starvation) ± VPS34i for 18 h.

Source Data Fig. 8 Source proteomics data for Fig. 8: Proteomics data (copies per cell) from naive CD8 T cells ± VPS34i for 5 h.

## Data Availability

The mass spectrometry proteomics data have been deposited to the ProteomeXchange Consortium via the PRIDE^84^ partner repository (https://www.ebi.ac.uk/pride) with dataset identifier PXD052729 for IL-7-maintained cells and PXD052733 for CTLs. The amino acid and VPS34 regulated CTL proteomes and VPS34 regulated naive CD8 proteomes are freely available to interrogate and explore on the Immunological Proteome Resource (www.immpres.co.uk)^22^. Source data are provided with this paper.
