# Author Correction: Autophagy repression by antigen and cytokines shapes mitochondrial, migration and effector machinery in CD8 T cells

**DOI:** 10.1038/s41590-026-02433-6

**Published:** 2026-01-20

**Authors:** Linda V. Sinclair, Tom Youdale, Laura Spinelli, Milica Gakovic, Alistair J. Langlands, Shalini Pathak, Andrew J. M. Howden, Ian G. Ganley, Doreen A. Cantrell

**Affiliations:** 1 Division of Cell Signalling and Immunology, School of Life Sciences, University of Dundee, Dundee, UK; 2 National Phenotypic Screening Centre, School of Life Sciences, University of Dundee, Dundee, UK; 3 MRC PPU, School of Life Sciences, University of Dundee, Dundee, UK

**Keywords:** Cytotoxic T cells, Autophagy

Correction to: *Nature Immunology* 10.1038/s41590-025-02090-1, published online 27 February 2025.

In the version of this article initially published, there was a typographical error in the units listed for the inhibitor VPS34i, where in the second paragraph of the Materials and Methods “Mice and cells” section, first paragraph of the “CTL killing assay” section, and in the first paragraph of the Reporting Summary “Sample preparation” section, “VPS34i (1 μM)” originally appeared as “VPS34i (1 nM).” The error has been corrected in the HTML and PDF versions of the article.

