# Supplementary material for: Autophagy repression by antigen and cytokines shapes mitochondrial, migration and effector machinery in CD8 T cells
Source: Nat Immunol. 2025 Feb 27;26(3):429–43. doi: 10.1038/s41590-025-02090-1 (PMC11876071; doi:10.1038/s41590-025-02090-1)
Supplement: Supplementary file 1 — Reporting Summary [file 41590_2025_2090_MOESM1_ESM.pdf]

Corresponding author(s): Linda Sinclair
Last updated by author(s): Jan 14, 2025

Reporting Summary

Nature Portfolio wishes to improve the reproducibility of the work that we publish. This form provides structure for consistency and transparency in reporting. For further information on Nature Portfolio policies, see our Editorial Policies and the Editorial Policy Checklist.

Statistics

For all statistical analyses, confirm that the following items are present in the figure legend, table legend, main text, or Methods section.

n/a	Confirmed	
☐	☒	The exact sample size (n) for each experimental group/condition, given as a discrete number and unit of measurement
☒	☐	A statement on whether measurements were taken from distinct samples or whether the same sample was measured repeatedly
☐	☒	The statistical test(s) used AND whether they are one- or two-sided<br>*Only common tests should be described solely by name; describe more complex techniques in the Methods section.*
☐	☒	A description of all covariates tested
☐	☒	A description of any assumptions or corrections, such as tests of normality and adjustment for multiple comparisons
☐	☒	A full description of the statistical parameters including central tendency (e.g. means) or other basic estimates (e.g. regression coefficient) AND variation (e.g. standard deviation) or associated estimates of uncertainty (e.g. confidence intervals)
☐	☒	For null hypothesis testing, the test statistic (e.g. F, t, r) with confidence intervals, effect sizes, degrees of freedom and P value noted<br>*Give P values as exact values whenever suitable.*
☒	☐	For Bayesian analysis, information on the choice of priors and Markov chain Monte Carlo settings
☒	☐	For hierarchical and complex designs, identification of the appropriate level for tests and full reporting of outcomes
☒	☐	Estimates of effect sizes (e.g. Cohen's d, Pearson's r), indicating how they were calculated

Our web collection on statistics for biologists contains articles on many of the points above.

Software and code

Policy information about availability of computer code

Data collection	Flow cytometry was performed on Novocyte (Agilent), FACSVerse or LSRFortessa (BD Biosciences) flow cytometers. FlowJo analysis software V10.9 and V10.10 (Becton Dickinson) was used to analyse all flow cytometry experiments. Target cell death experiments were analysed with an iQue Screener (Sartorius). The oxygen consumption rate (OCR) and extracellular acidification rate (ECAR) of CTL were measured using a Seahorse XF24 analyser.<br>Proteomics data were acquired on a Q Exactive HF-X (Thermo Scientific) mass spectrometer coupled with a Dionex Ultimate 3000 RS (Thermo Scientific)
Data analysis	FlowJo analysis software V10.9 and V10.10 (Becton Dickinson) was used to analyse all flow cytometry experiments.<br>Raw DIA mass spec data files were searched using Spectronaut version 16.0.220606.53000 (Biognosys). P-values and fold changes for volcano plots were calculated using RStudio (v 2023.06.1+524) with the Bioconductor package “limma” (v3.54.2) .<br>Heat maps were generated using the Morpheus tool from the Broad Institute (https://software.broadinstitute.org/morpheus). Statistical analyses were performed using Prism 10, GraphPad Software.

For manuscripts utilizing custom algorithms or software that are central to the research but not yet described in published literature, software must be made available to editors and reviewers. We strongly encourage code deposition in a community repository (e.g. GitHub). See the Nature Portfolio guidelines for submitting code & software for further information.

Data

Policy information about availability of data

All manuscripts must include a data availability statement. This statement should provide the following information, where applicable:

- Accession codes, unique identifiers, or web links for publicly available datasets
- A description of any restrictions on data availability
- For clinical datasets or third party data, please ensure that the statement adheres to our policy

The mass spectrometry proteomics data have been deposited to the ProteomeXchange Consortium via the PRIDE partner repository (https://www.ebi.ac.uk/pride/) with the dataset identifier PXD052729 for IL7 maintained cells and PDX052733 for CTL.

Research involving human participants, their data, or biological material

Policy information about studies with human participants or human data. See also policy information about sex, gender (identity/presentation), and sexual orientation and race, ethnicity and racism.

Reporting on sex and gender	not applicable
Reporting on race, ethnicity, or other socially relevant groupings	not applicable
Population characteristics	not applicable
Recruitment	not applicable
Ethics oversight	not applicable

Note that full information on the approval of the study protocol must also be provided in the manuscript.

Field-specific reporting

Please select the one below that is the best fit for your research. If you are not sure, read the appropriate sections before making your selection.

☒ Life sciences ☐ Behavioural & social sciences ☐ Ecological, evolutionary & environmental sciences

For a reference copy of the document with all sections, see nature.com/documents/nr-reporting-summary-flat.pdf

Life sciences study design

All studies must disclose on these points even when the disclosure is negative.

Sample size	Group sizes for animal studies were determined by previous pilot studies and experience with regard to the experimental parameters measured.
Data exclusions	No data were excluded
Replication	In vitro experiments were replicated a minimum of 3 times to verify reproducibility.
Randomization	no randomization was used. Randomization was not possible for this study. Where cells were treated with inhibitors, or amino acid deprivation, the control and treated cells came from the same starting pool of cells.
Blinding	no blinding was performed. T cell populations were analyzed at defined stages in activation and differentiation and blinding the samples would have been challenging. The proteomics approach is unbiased, and statistics were performed (as detailed in the text) to identify significant changes in protein abundance.

Reporting for specific materials, systems and methods

We require information from authors about some types of materials, experimental systems and methods used in many studies. Here, indicate whether each material, system or method listed is relevant to your study. If you are not sure if a list item applies to your research, read the appropriate section before selecting a response.

Materials & experimental systems

n/a	Involved in the study	
☐	☒	Antibodies
☐	☒	Eukaryotic cell lines
☒	☐	Palaeontology and archaeology
☐	☒	Animals and other organisms
☒	☐	Clinical data
☒	☐	Dual use research of concern
☒	☐	Plants

Methods

n/a	Involved in the study	
☒	☐	ChIP-seq
☐	☒	Flow cytometry
☒	☐	MRI-based neuroimaging

Antibodies

Antibodies used	FC receptors were blocked using FC Block (BD Pharmingen Cat # 553141) at 1 ug /million cells. The following antibodies were used for flow cytometry:<br>Cell surface staining antibody clones used were: CD8a (53-6.7), CD44 (IM7), IL-7Ra (A7R34), CD62L (MEL-14). Antibodies conjugated to APC, AlexaFluor 647, PeCy7, PerCPCy5.5, APC-efluor780, Brilliant Violet 421 and 605 were obtained from either eBioscience, Biolegend or BD Biosciences.<br>Cell surface staining antibodies were used at a final dilution 1:200.<br>For intracellular staining: phospho S6 Ser235/236 (cat no. 2211; Cell Signaling Technology) or phospho ACC S79 (clone D7D11 cat no. 11818;Cell Signaling Technology) were used at 1:100 dilution. Anti-rabbit Alexa 647 was used as the secondary, detection stain (1:100; cat no. 4414; Cell Signaling technology).
Validation	Validation of the specificity of these antibodies can be found at the manufacturers websites.

Eukaryotic cell lines

Policy information about cell lines and Sex and Gender in Research

Cell line source(s)	The MC38 colon cancer cell line was purchased from Kerafast.<br>E.G7-OVA (CRL-2113) were obtained ATCC.
Authentication	MC38 cell line authentication data: https://www.kerafast.com/PDF/KF_STR_MC38_ENH204FP.pdf<br>E.G7-OVA (CRL-2113) were directly obtained commercially from ATCC where these are routinely authenticated.
Mycoplasma contamination	Fresh MC38 cells were brought up from frozen (manufacturers) stock vials (which are routinely tested for mycoplamsa) and used for tumour engraftment after 3 passages. E.G7-OVA (CRL-2113) are routinely tested for mycoplasma.
Commonly misidentified lines<br>(See ICLAC register)	No commonly misidentified lines were used

Animals and other research organisms

Policy information about studies involving animals; ARRIVE guidelines recommended for reporting animal research, and Sex and Gender in Research

Laboratory animals	OT-1 mice (Charles river UK, stock number 003831)(Hogquist et al. 1994);<br>autophagy reporter mice (McWilliams et al. 2019);<br>mitophagy reporter/mito-QC transgenic mice (McWilliams et al. 2016; McWilliams et al. 2018; McWilliams et al. 2019);<br>CD4CreAMPKa1fl/fl (Rolf, J. et al. 2013).<br>C57BL/6 (wild-type, WT; Charles River) mice.<br>All mice were used at 8-12 weeks of age.
Wild animals	This study did not involve wild animals.
Reporting on sex	Both male and female mice were used to generate in vitro activated CD8 T cells. There were no observed phenotypic differences (in autophagy flux) attributable to sex. Both male and female mice were used for rListeria infection studies. There were no observed phenotypic differences attributable to sex. Female mice were used for MC38 tumour model.
Field-collected samples	No field-collected samples were used in this study
Ethics oversight	All animal procedures were performed under approval from the University of Dundee Ethical Review Process and according to UK Home Office regulations and the ARRIVE 2.0 guidelines. Mice were bred and maintained in the WTB/RUTG, University of Dundee in compliance with UK Home Office Animals (Scientific Procedures) Act 1986 guidelines. Mice were group-housed according to litter size with food and water available ad libitum, on a 12:12h light: dark schedule and maintained at constant temperature (20±1o C) and humidity.

Note that full information on the approval of the study protocol must also be provided in the manuscript.

Plants

Seed stocks: not applicable

Novel plant genotypes: not applicable

Authentication: not applicable

Flow Cytometry

Plots

Confirm that:

☒ The axis labels state the marker and fluorochrome used (e.g. CD4-FITC).

☒ The axis scales are clearly visible. Include numbers along axes only for bottom left plot of group (a 'group' is an analysis of identical markers).

☒ All plots are contour plots with outliers or pseudocolor plots.

☒ A numerical value for number of cells or percentage (with statistics) is provided.

Methodology

Sample preparation

T-cells were cultured in RPMI 1640 containing glutamine (Gibco), supplemented with 10 % FBS (Gibco), 1 % penicillin/streptomycin (Gibco) and 50 µM β-mercaptoethanol(Sigma) (RPMI 1640 complete media) at 37oC and 5% CO2. Single cell suspensions were generated by disaggregation of lymph nodes or spleens. Splenic red blood cells were lysed in ACK buffer (150 mM NH4Cl, 10 mM KHCO3, 110 µM Na2EDTA, pH 7.8). Cell suspensions were washed in complete culture medium prior to activation. To maintain naïve T-cells, splenic and lymph node single cell suspensions were cultured with Interleukin 7 (IL-7) (5 ng/ml, PeproTech) in complete RPMI 1640 medium. VPS34-IN138 (VPS34i;1µM), Halofuginone (100nM), Bafilomycin A1 (200nM), ruxolitinib (1µM) or rapamycin (20nM) were used where indicated.

The EasySep Mouse CD8+ T Cell Isolation Kit (STEMCell technologies) was used to purify naïve OT-1 CD8+ T cells for proteomic analysis.

Polyclonal T-cell activation:
To generate cytotoxic T lymphocytes (CTL), cells were stimulated with 1µg/ml of CD3 antibody (2C11, BioLegend) and 2µg/ml CD28 antibody (37.51, ebiosciences) in RPMI 1640 complete media for 48hrs. Cells were then washed out of activation conditions and polyclonally expanded in RPMI 1640 complete media supplemented with IL-2 (20ng/ml, Proleukin, Novartis) at a density of 3x105 cells/ml for a further 3–5 days.

OT-I TCR transgenic T-cell activation:
For generation of OT-1 CTL, lymph nodes single cell suspensions were isolated from OT-1 TCR transgenic mice and stimulated for 36 hours with SIINFEKL peptide (10ng/ml). Cells were washed out of activation conditions and clonally expanded in RPMI 1640 complete media supplemented with IL-2 (20ng/ml, Proleukin, Novartis) at a density of 3x105 cells/ml for a further 3–5 days.
For experiments with IL-12 and IL-18, activated OT-1 T-cells were initially expanded in IL-2 (20ng/ml) and IL-12 (20ng/ml; Peprotech) before being switched into IL-2 alone, IL-12 + IL-18 (20ng/ml; R&D Systems) or no cytokine for 24h. For experiments with IL-15, 36h SIINFEKL activated OT-1 T-cells were expanded in IL-15 (20ng/ml; Peprotech).

Nutrient deprivation studies:
Prior to nutrient deprivation, cells were washed twice in PBS. Cells were then resuspended at 3x105 cells/ml in either complete RPMI or nutrient deprivation medium listed below. All nutrient deprivation media were supplemented with 10 % Dialysed FBS (dFCS) (Gibco), 1% penicillin/streptomycin (Gibco) and 50 µM β-mercaptoethanol (Sigma).
• For total amino acid starvation cells were cultured in Hanks' Balanced Salt solution (HBSS, Gibco).
• For glucose starvation cells were cultured in RPMI 1640 media lacking glucose (Gibco).
• For glutamine starvation cells were cultured in RPMI 1640 media lacking glutamine (Gibco).
• For arginine or methionine starvation cells were cultured in RPMI 1640 media lacking arginine, lysine and methionine (DC Biosciences). Supplementation of individual amino acids (Sigma) to the levels of standard RPMI 1640 formula was carried out to generate culture medium lacking a single amino acid, methionine or arginine.

Flow cytometry sample acquisition and analysis:
•For surface staining -antibody clones used were: CD8a (53-6.7), CD44 (IM7), IL-7Ra (A7R34), CD62L (MEL-14), Fc Block (BD Biosciences; Cat # 55314, 1 ug/million cells). Antibodies conjugated to APC, AlexaFluor 647, PeCy7, PerCPCy5.5, APC-efluor780, Brilliant Violet 421 and 605 were obtained from either eBioscience, Biolegend or BD Biosciences. For cell surface staining, 1:200 dilution of staining antibodies was used. For experiments with live/dead determination, DAPI (4',6-

diamidino-2-phenylindole, Thermo) was used as a viability indicator and added at 250ng/ml prior to acquisition.
•For intracellular staining, cells were fixed with 1% formaldehyde (v/v) prior to permeabilization with 90% (v/v) ice cold methanol for 30 mins. Cells were washed and incubated with antibody against phospho S6 Ser235/236 (cat no. 2211; 1:100) or phospho ACC S79 (clone D7D11 cat no. 11818; 1:100) prior to incubation with anti-rabbit Alexa 647 secondary (cat no. 4414; 1:100; all Cell Signaling technology). Cells were washed and resuspended in 0.5% FBS/PBS (v/v) for flow cytometric acquisition.
•Mitotracker Deep Red (cat no. M22426) and MitoSOX (cat no. M36008) staining was performed according to manufacturer's guidelines (Invitrogen, Thermo Scientific).
•Kynurenine uptake to monitor System L transporter activity was done according to established protocols (Sinclair, L. V. et al 2018) . Briefly, surface-stained cells were incubated in pre-warmed HBSS (GIBCO) with 200uM kynurenine (Sigma) for 4 min at 37C prior to fixation with 1% formaldehyde (v/v). Where indicated kynurenine uptake was performed with 10mM BCH (Sigma), a competitive inhibitor for System L uptake. Cells were washed post-fixation and resuspended in 0.5% FBS/PBS for flow cytometric acquisition. Kynurenine uptake was detected by emission at 450nm.

•SLC1A5-mediated uptake capacity was measured by using azide-substrate and copper-click labelled to a fluorophore for detection as described in (Pelrom L.R. et al, 2023). Briefly, cells were incubated in pre-warmed HBSS (GIBCO) with 100uM Azidohomoalanine (AHA; Cambridge reserach) for 4 min at 37C prior to fixation with 1% formaldehyde (v/v). Cells were permeabilised with 0.01% saponin (Sigma) in PBS for 20 mins prior to incubation with the click-mixture containing AZDye 647 Alkyne (Click Chemistry tools).

•Flow cytometric measurements of autophagy and mitophagy flux
An autophagy flux reporter comprising an mCherry:GFP:Map1lc3b (mCherry:GFP:LC3b) fusion protein expressed ubiquitously from the ROSA-26 locus was used. Autophagic flux can be quantified by analysing GFP fluorescence quenching normalized to mCherry signals. Autophagy flux is measured by calculating the mCherry / GFP fluorescence ratio as a 'derived parameter' in FlowJo v10. mCherry/GFP values of 1 indicate low autophagy; mCherry/GFP values above 1 indicate high autophagy flux.
A mitophagy flux reporter comprising an mCherry-GFP-mtFIS1 fusion protein expressed ubiquitously from the ROSA-26 locus was also used (McWilliams, T. G. et al. 2019 ; McWilliams, T. G. et al. 2018). Mitophagy flux is measured by calculating the mCherry / GFP fluorescence ratio as a 'derived parameter' in FlowJo v10.

Instrument	Flow cytometry was performed on Novocyte (Agilent), FACSVerse or LSRFortessa (BD Biosciences) flow cytometers.
Software	FlowJo analysis software V10.9 and V10.10 (Becton Dickinson) was used to analyse all flow cytometry experiments.
Cell population abundance	The EasySep Mouse CD8+ T Cell Isolation Kit (STEMCell technologies) was used to purify naïve OT-1 CD8+ T cells for proteomic analysis. Cell purity was determined by flow cytometry analysis of post-isolation samples and purity was >90% .
Gating strategy	During acquisition, lymphocytes were identified by gating on forward scatter area (FSC-A) and side scatter area (SSC-A). Doublets were excluded using FSC-A vs forward scatter width (FSC-W). In experiments to determine viability, dead cells were determined based on DAPI (4',6-diamidino-2-phenylindole, 0.5 ug/mL; Life Technologies) staining. Specific experimental gating strategies are referred to in figures .

☒ Tick this box to confirm that a figure exemplifying the gating strategy is provided in the Supplementary Information.
